# Chemical Constituents, Pharmacological Activities, and Cardiovascular Protective Mechanisms of *Dendrobium* Species: A Review

**DOI:** 10.3390/ijms27094149

**Published:** 2026-05-06

**Authors:** Yue Hu, Zhiyong Li, Jian Li, Xiaowen Li, Meina Wang

**Affiliations:** 1 The Orchid Conservation & Research Center of Shenzhen and the National Orchid Conservation Center of China, Shenzhen Key Laboratory for Orchid Conservation and Utilization, Key Laboratory of National Forestry and Grassland Administration on *Dendrobium officinale*, Shenzhen 518114, China; huyuea0914@126.com (Y.H.); lizhiyong83@hotmail.com (Z.L.); etecology@foxmail.com (J.L.); shelwin525@163.com (X.L.); 2 School of Biological Sciences and Technology, Beijing Forestry University, Beijing 100083, China

**Keywords:** *Dendrobium*, chemical components, chemodiversity, pharmacological activity, cardiovascular diseases

## Abstract

The genus *Dendrobium*, a well-known traditional Chinese medicinal herb, contains complex and diverse chemical constituents. The plant has been widely used in traditional medicine and has attracted increasing attention in modern pharmacological research due to its therapeutic potential. Bibliographic searches were conducted across various recognized databases. The exploration covered the years 1965–2025, and the connectors ‘and’ and ‘or’ were used with keywords such as “*Dendrobium*”, “phytochemistry”, “pharmacology”, “Cardiovascular diseases”, and “extracts”. The chemical composition of the genus *Dendrobium* mainly includes alkaloids, bibenzyls, flavonoids, phenanthrenes, phenylpropanoids, and terpenoids. Modern pharmacological studies have demonstrated that the genus *Dendrobium* exhibits multiple biological effects, including anti-tumor, antibacterial, anti-inflammatory, hypoglycemic, and neuroprotective activities. Notably, the genus *Dendrobium* shows significant potential in cardiovascular disease prevention and treatment through mechanisms such as antioxidant stress, anti-inflammation, regulation of lipid metabolism, anti-atherosclerosis, and inhibition of myocardial fibrosis. This review provides a comprehensive overview of the chemical components and pharmacological activities of *Dendrobium* plants, with emphasis on their cardiovascular protective effects. These findings offer a scientific basis for the further development and clinical application of *Dendrobium* medicinal resources.

## 1. Introduction

Cardiovascular diseases (CVDs) are the leading cause of death and disability worldwide [1,2], and their pathological mechanisms involve multiple aspects such as oxidative stress, lipid metabolism disorders, inflammatory responses, and myocardial fibrosis [3]. According to data from the World Health Organization (WHO), as of 2023, there were approximately 626 million patients with CVDs globally, and the related death toll was approximately 1.92 billion [4]. In China, the incidence and mortality rates of CVDs remain at a high level. According to the “2024 China Cardiovascular Health and Disease Report”, the number of deaths due to CVDs has accounted for over 40% of the total deaths among the population [5]. Given the significant disease burden, exploring effective prevention and treatment strategies for CVDs has become an urgent topic in modern medical research.

Among the numerous potential therapeutic resources in traditional Chinese medicine, *Dendrobium* demonstrates unique value. This work provides a systematic review covering the entire *Dendrobium* genus—the second largest genus in the Orchidaceae, comprising approximately 1500 species and exhibiting a wide distribution across Asia, Europe, and Australia [6,7]. There are nearly 80 species of *Dendrobium* in China, predominantly found in South China and Southwest China [8]. Botanically, the medicinal species of the genus *Dendrobium* are mostly perennial epiphytic herbs, with fleshy and erect or pendulous stems, which are the main medicinal parts. There are significant interspecific differences in botanical traits, chemical constituent types and content among different species of the genus: the stems vary in shape from cylindrical and fusiform to clavate; the core secondary metabolites are significantly different among species, with some species rich in alkaloids, some dominated by polysaccharides, and others characterized by bibenzyls and phenanthrenes, which leads to differences in their pharmacological activity profiles. As a critical component of traditional Chinese medicine, *Dendrobium* was classified as a premium medicinal herb in *Shennong Bencao Jing* [9,10]. *Dendrobium* species such as *Dendrobium officinale* Kimura & Migo, *Dendrobium nobile* Lindl., *Dendrobium huoshanense* Z.Z.Tang & S.J.Cheng, *Dendrobium chrysotoxum* Lindl., and *Dendrobium fimbriatum* HooK. are officially recognized in the Chinese Pharmacopoeia (2020 Edition) [11]. The chemical components and pharmacological activities of *Dendrobium* have always been a research hotspot in the fields of medicine and botany.

Modern research has shown that *Dendrobium* contains various active components such as polysaccharides, alkaloids, and bibenzyls, which possess multiple pharmacological effects including antioxidant, anti-inflammatory, and lipid metabolism regulation [12,13,14,15]. This has led to increasing attention in the field of CVD prevention and treatment. This review aims to systematically summarize the chemical components and pharmacological effects of the plants in the genus *Dendrobium*, with a focus on the intervention effects and research progress on key pathological links in CVDs (such as oxidative stress, lipid metabolism, atherosclerosis formation, inflammation, and fibrosis). By integrating existing evidence, it is expected to provide a systematic theoretical reference and directional guidance for in-depth research and clinical application of *Dendrobium* in CVD prevention and treatment.

Compared with the existing reviews, the innovation and uniqueness of this review mainly lie in the following three aspects: (1) this review systematically summarizes the diversity of chemical metabolites in the *Dendrobium* genus and, in combination with the latest research progress up to 2025, analyzes the pharmacological potential of different core metabolic substance skeletons; (2) this review focuses on the cardiovascular protective effects of the *Dendrobium* genus and comprehensively elaborates on the intervention effects and molecular mechanisms of its active metabolites on all key pathological links of cardiovascular diseases (including oxidative stress, lipid metabolism disorders, atherosclerosis, inflammation, myocardial fibrosis, and hypertension); (3) this review systematically summarizes the current challenges and limitations of the clinical translation of *Dendrobium* in the field of cardiovascular diseases, and proposes specific future research directions, providing systematic theoretical references for in-depth research and clinical application of the medicinal resources of *Dendrobium* in the prevention and treatment of cardiovascular diseases. The complete source information of all compounds from different *Dendrobium* species, as well as the interspecific chemodiversity data, are fully detailed in Table S1 of the Supplementary Materials.

## 2. Main Chemical Components and Physiological Activities of Dendrobium

### 2.1. Alkaloids

**Figure 1:**
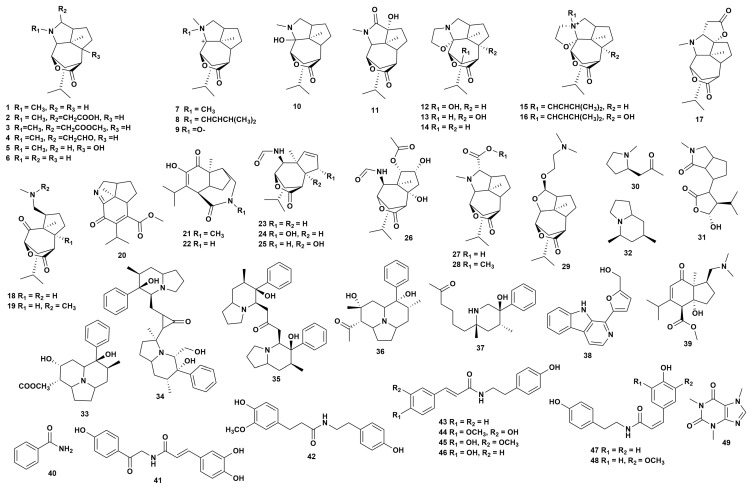
Structure of alkaloids in *Dendrobium*.

Based on their distinct chemical structures, seven categories of alkaloids have been identified in the *Dendrobium* genus, namely sesquiterpenes, pyrrolidines, indolizidines, piperidines, indoles, organic amines, and purines (Figure 1, Table S1). With 29 derivatives found in four *Dendrobium* species, sesquiterpenoid alkaloids (**1**–**29**), which only contain a picrotoxane skeleton, exhibit the greatest structural diversity of these classes [16,17,18,19,20,21,22,23,24,25,26,27,28,29,30,31]. Pyrrolidine alkaloids have a parent nucleus comprising one or two pyrrolidine rings, with two compounds (**30**–**31**) identified across three *Dendrobium* species [21,32,33]. Indolizidine alkaloids are structurally composed of piperidine and pyrrolidine rings, with five compounds (**32**–**36**) isolated from three *Dendrobium* species [32,33,34,35,36]. The piperidine alkaloid class is characterized by a piperidine ring, with one compound (**37**) isolated exclusively from *Dendrobium Crepidatum* Lindl. & Paxton [36]. Only compound **38** from *D. huoshanense* represents the indole alkaloid class [37]. Organic amine alkaloids exhibit a relatively simple structure, lacking nitrogen atoms within a ring, with 10 compounds (**39**–**48**) isolated from eight *Dendrobium* species [32,38,39,40,41,42,43,44,45,46,47]. Purine alkaloids are still species-specific; compound **49** was exclusively isolated from *Dendrobium aphyllum* (Roxb.) C.E.C.Fisch. (**49**) [48].

Benzodiazepine compounds are the active components of the genus *Dendrobium* and possess various biological activities and medicinal values. For instance, the total alkaloids from *D. crepidatum* can exert a protective effect against acute lung injury by down-regulating the TLR4-mediated MyD88/MAPK signaling pathway [49]. Indole–rhizine-type alkaloids (**35**) and piperidine-type alkaloids (**37**) [36] exhibit strong inhibitory effects on NO release, and can to some extent suppress the release of NO by LPS-induced RAW264.7 macrophages.

### 2.2. Bibenzyls

**Figure 2:**
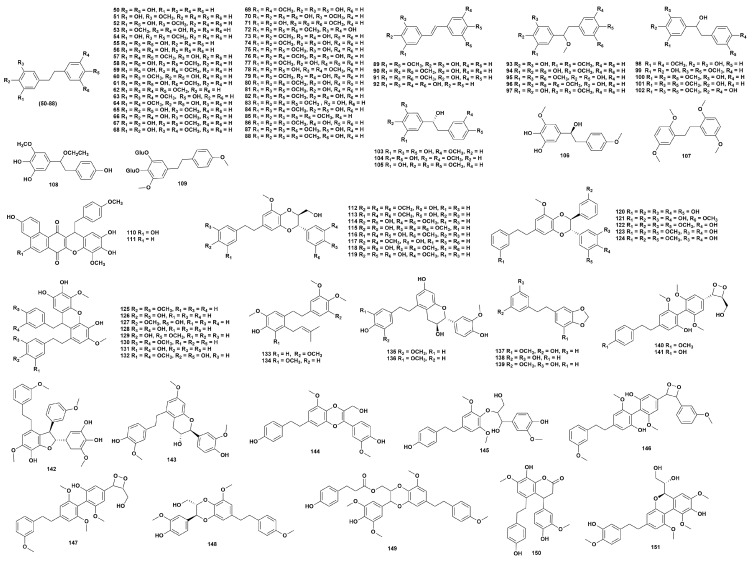
Structure of bibenzyl compounds in *Dendrobium*.

Bibenzyls are a class of compounds with a 1,2-diphenylethane skeleton, and are widely distributed in the plants of the genus *Dendrobium*. Currently, 102 bibenzyl compounds (**50**–**151**) have been isolated from 32 species of *Dendrobium* [16,25,28,31,35,37,42,43,44,50,51,52,53,54,55,56,57,58,59,60,61,62,63,64,65,66,67,68,69,70,71,72,73,74,75,76,77,78,79,80,81,82,83,84,85,86,87,88,89,90,91,92,93,94,95,96,97,98,99,100,101] (Figure 2, Table S1).

The bibenzyl compounds found in the genus *Dendrobium* have shown great potential in modern pharmacological research. Studies have shown that the bibenzyls extracted and isolated from the *Dendrobium* plants have excellent anti-tumor, anti-diabetic, and neuroprotective effects [14]. Through the method of network pharmacology, the key compounds and targets in *Dendrobium* were identified. Among them, compounds **58**, **83**, and **84** can exert anti-liver cancer effects through multiple targets and pathways [102]. Compounds **51** and **56** exhibit antibacterial activity against *Bipolaris oryzae* [51]. Boonchoo et al. [103] found that compound **69** can inhibit the metastasis of lung cancer cells (H292) by inducing apoptosis.

### 2.3. Flavonoids

**Figure 3:**
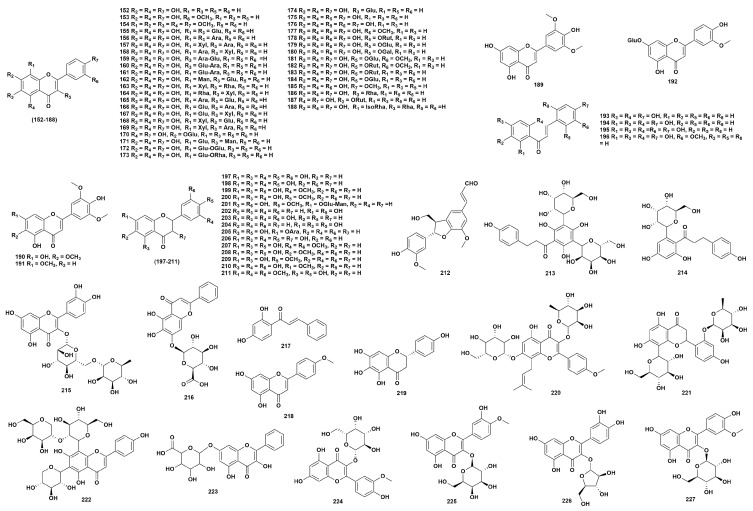
Structure of flavonoids in *Dendrobium*.

Flavonoids are one of the most abundant natural products in the plantae. Currently, 76 flavonoids (**152**–**227**) have been isolated from 17 species of the genus *Dendrobium* (Figure 3, Table S1) [16,32,43,54,56,57,59,61,62,63,65,66,68,69,77,79,85,92,104,105,106,107,108,109,110,111,112,113]. Flavonoid compounds possess a variety of pharmacological activities such as antibacterial, antiviral, anti-diabetic, anti-inflammatory and anti-cancer properties [114]. Wang et al. [104] demonstrated that compounds **152**, **156**, **162**, **174**, **186** and **188** have certain neuroprotective effects against corticosterone-induced damage to PC12 cells. Feng et al. [106] showed that compounds **162**, **165**, **166**, **171** and **188** have significant α-glucosidase inhibitory activity and are expected to be used as lead compounds for the development of hypoglycemic drugs. Chi et al. [112] demonstrated that compound **204** has certain α-glucosidase inhibitory activity, with an IC_50_ value of 98.95 ± 2.53 μmol/L.

### 2.4. Phenolic Compounds

**Figure 4:**
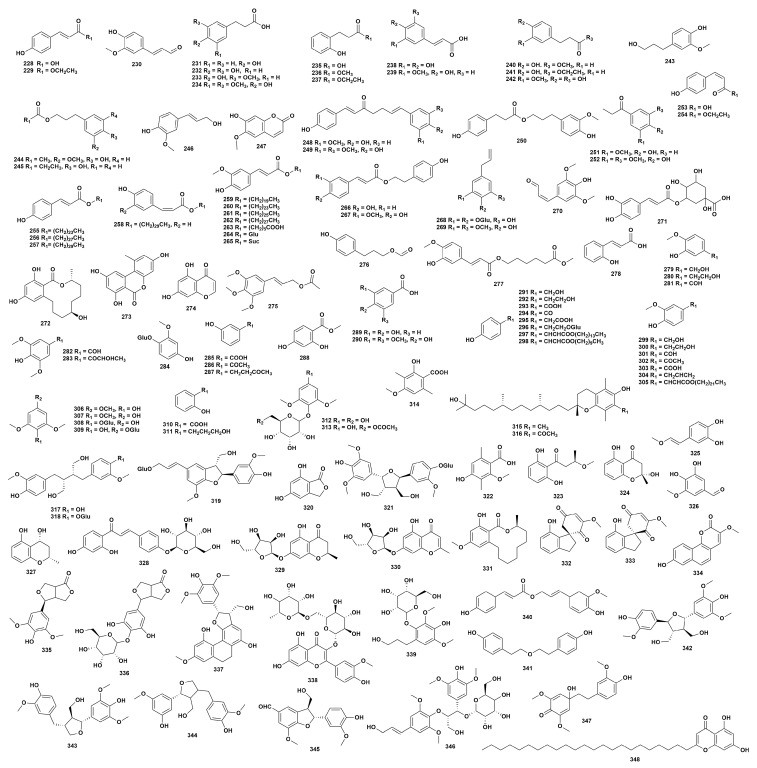
Structure of phenolic compounds in *Dendrobium*.

Phenolic compounds are organic compounds that have one or more hydroxyl groups directly attached to the benzene ring. Phenolic compounds are widely present in the genus *Dendrobium*. Currently, 121 phenolic compounds (**228**–**348**) have been isolated from 29 species of *Dendrobium* plants (Figure 4, Table S1) [16,25,27,32,37,41,42,43,48,52,56,58,60,61,64,65,67,68,70,77,78,79,80,81,82,85,87,95,99,105,107,109,112,113,115,116,117,118,119,120,121,122,123,124,125,126,127,128,129,130,131,132,133,134,135,136,137]. Studies have shown that phenolic compounds in *Dendrobium* possess various pharmacological activities such as antibacterial, antioxidant and anti-tumor properties. Zhang et al. [138] found that compounds **228** and **250** have inhibitory activity against *Ralstonia solanaceanum*, and compound **301** has inhibitory activity against *Staphylococcus aureus*. Chi et al. [112] demonstrated that compound **248** exhibited α-glucosidase inhibitory activity, with an IC_50_ of 65.60 ± 3.31 μmol/L. Compound **316** exhibits significant antioxidant activity and has inhibitory effects on HepG2 cells (IC_50_ = 51.28 ± 3 μmol/L) and Hela cells (IC_50_ = 18.71 ± 3 μmol/L) [27]. Guo et al. [82] found that compound **343** has significant tyrosinase inhibitory activity, with an IC_50_ of 10.40 ± 0.36 μmol/L.

### 2.5. Simple Phenylpropanoids

**Figure 5:**
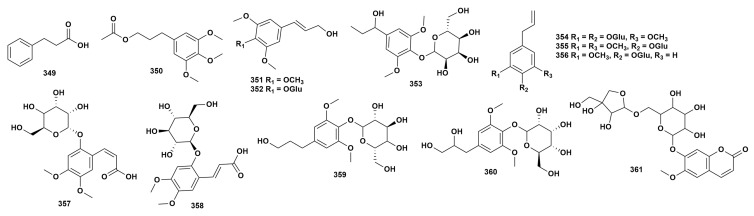
Structure of simple phenylpropanoids in *Dendrobium*.

The skeleton of simple phenylpropanoids consists of a benzene ring and three straight-chain carbon atoms (the C_6_–C_3_ group). Currently, 13 simple phenylpropanoid compounds (**349**–**361**) have been isolated from five species of the genus *Dendrobium* (Figure 5, Table S1) [37,41,49,65,121,129,136,137]. The phenylpropanoid components are a very important class of natural organic compounds in nature, possessing anti-inflammatory, antioxidant, and anti-tumor activities [139]. Among the plants of *Dendrobium*, only a limited number of simple phenylpropanoids have been isolated, and the related pharmacological activity studies are also relatively few.

### 2.6. Coumarins

**Figure 6:**
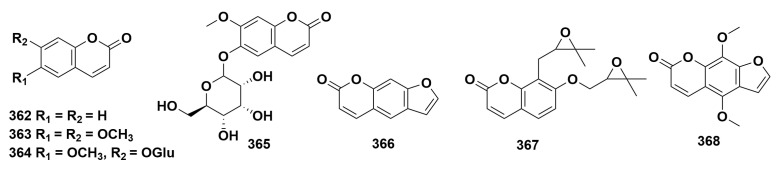
Structure of coumarins in *Dendrobium*.

Coumarins isolated from species of the genus *Dendrobium* always present in the core benzo-α-pyrranone scaffold, while site-specific substitutions lead to structural diversification (Figure 6, Table S1). Across ten *Dendrobium* species, seven coumarin derivatives (**362**–**368**) have been found [16,41,52,73,80,95,121,123,125,128,130]. Coumarins are a class of important natural organic substances, possessing anti-inflammatory, anti-tumor and antioxidant activities [140]. Currently, there are relatively few studies on the activity of coumarin compounds in *Dendrobium*. Shi et al. [141] determined the content of coumarins (such as coumarin and scoparone) in eight species of *Dendrobium*, and the results showed that the types and contents of coumarin compounds in different species of *Dendrobium* plants vary significantly.

### 2.7. Lignans

**Figure 7:**
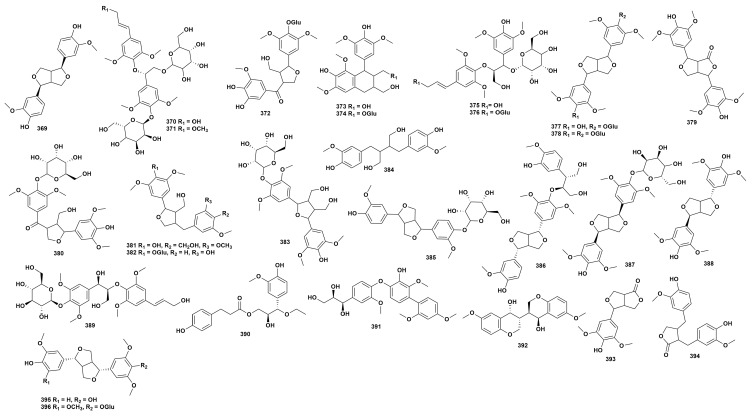
Structure of lignans in *Dendrobium*.

Lignans are natural compounds synthesized from two phenylpropyl C_6_-C_3_ structural units, exhibiting structural diversity through stereochemical variation, glycosylation, and oxidative cyclization. Currently, 28 lignans have been isolated from 14 species of *Dendrobium* (**369**–**396**). The chemical structure of these lignans is depicted in Figure 7, while the source information for the compounds is detailed in Table S1 [16,37,41,43,63,65,68,70,80,82,99,100,109,118,120,130,134,136,137,142,143,144]. The lignans in the genus *Dendrobium* possess various pharmacological activities such as antioxidant, antibacterial and anti-tumor properties. Guo et al. [82] found that compound **369** has significant tyrosinase inhibitory activity, with an IC_50_ of 12.16 ± 0.18 μmol/L. Meng et al. [145] demonstrated that compound **383** has ABTS and DPPH free radical scavenging ability, showing certain antioxidant activity. Compound **391** exhibits good inhibitory activity against A549 cells, with an IC_50_ of 29.35 μM [134].

### 2.8. Quinonoids

**Figure 8:**
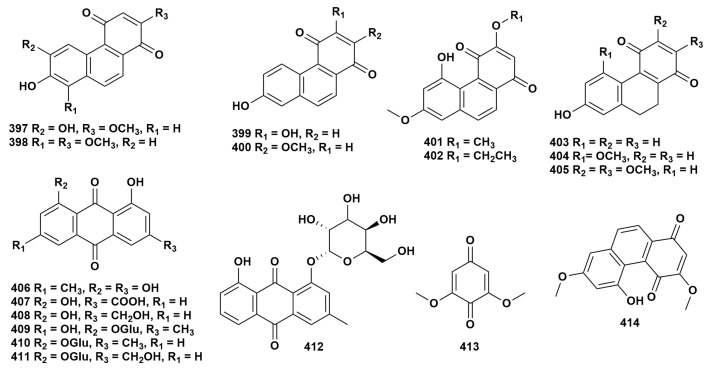
Structure of quinonoids in *Dendrobium*.

Quinones represent a class of aromatic organic compounds characterized by a dione structure with two double bonds within a six-carbon atom ring. Currently, 18 quinones have been isolated from nine species of *Dendrobium* (**397**–**414**) [16,31,41,48,51,68,75,77,86,91,121,128,143]. The chemical structure of these compounds is depicted in Figure 8, while Table S1 provides the source information for each compound. Research conducted by Liu et al. [146] demonstrated that compound **399** exhibited in vitro growth inhibitory activity against three types of tumor cells, namely HL-60, A549, and MCF-7. Zhang et al. [147] also found that compound **401** could inhibit the proliferation and metastasis of human ovarian cancer cells HO-8910PM by up-regulating the gene expressions of *CASP3*, *CASP9*, and *CAV1*, and down-regulating the expression of *SOX2*.

### 2.9. Phenanthrene Compounds

**Figure 9:**
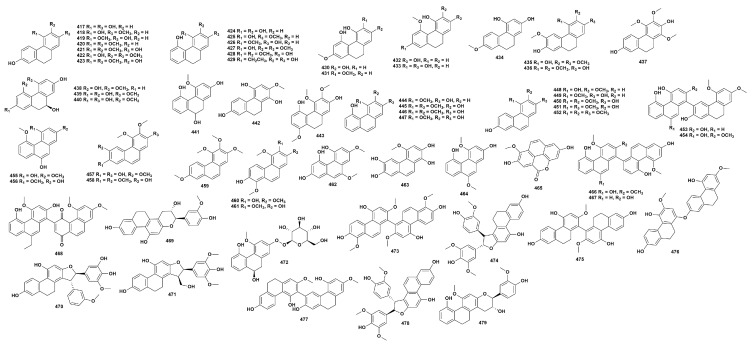
Structure of phenanthrene compounds in *Dendrobium*.

Phenanthrenoids are a class of fused polycyclic aromatic hydrocarbons characterized by a core structure consisting of three benzene rings with non-collinear centers, exhibiting structural diversity through hydroxylation, methoxylation, and dihydro modification (Figure 9, Table S1). To date, 65 phenanthrene-type compounds have been isolated from 25 *Dendrobium* species (**415**–**479**) [16,52,53,56,58,61,62,64,65,68,69,72,73,75,78,83,84,85,87,92,93,100,112,113,118,120,123,125,128,133,136,142,144,148,149]. The phenanthrenoids of the genus *Dendrobium* have pharmacological effects such as neuroprotection and hypoglycemic action. Compound **416** exhibits significant α-glucosidase inhibitory activity [83]. Zhang et al. [144] conducted research showing that compound **450** has certain inhibitory activity against acetylcholinesterase. Li et al. [62] conducted research indicating that compounds **415** and **469** have significant antioxidant and anti-inflammatory activities, and in addition, compound **469** can inhibit the activity of α-glucosidase. Compound **479** has the ability to inhibit the generation of NO, with an IC_50_ of 10.9 mM [12].

### 2.10. Terpenoids

**Figure 10:**
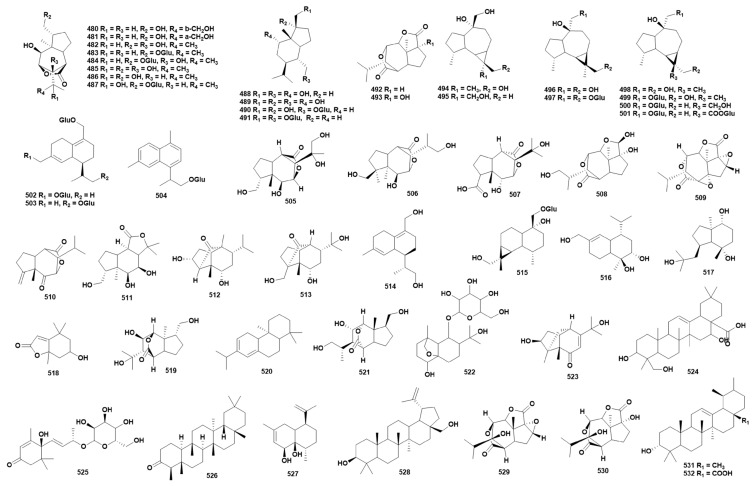
Structure of terpenoids in *Dendrobium*.

Terpenoids represent a class of linear polymers composed of isoprene units (C_5_H_8_). A total of 53 terpenoids have been isolated from 10 species of *Dendrobium* (**480**–**532**) [16,27,32,41,80,82,113,121,124,126,127,150,151]. The chemical structure of these compounds is shown in Figure 10, while Table S1 provides the source information for each compound. Studies have shown that compound **500** exhibits certain in vitro anti-inflammatory activity, with the IC_50_ of its inflammatory factor TNF-α being 14.39 ± 0.99 μM [152]. Compounds **490**, **491** and **500** have immunomodulatory activity [153].

### 2.11. Steroids

**Figure 11:**
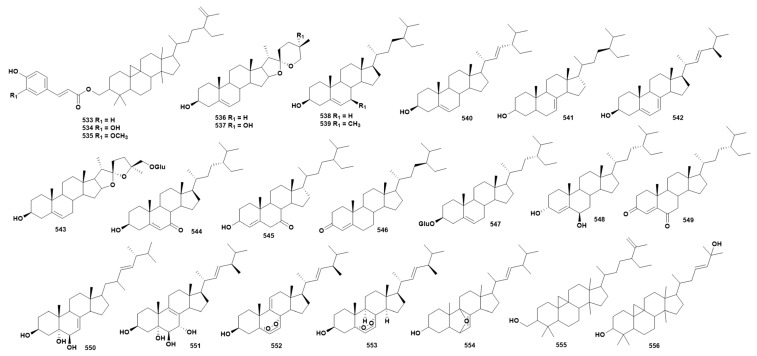
Structure of steroid compounds in *Dendrobium*.

Steroids in *Dendrobium* species universally possess the cyclopentanophenanthrene tetracyclic skeleton, and a total of 24 steroid compounds have been isolated from 18 species of *Dendrobium* (**533**–**556**) [16,27,41,57,63,65,69,79,80,86,93,94,105,112,113,117,118,123,124,125,131,133,134,151,154,155,156]. The chemical structure of these compounds is depicted in Figure 11, while Table S1 provides detailed source information. Research shows that compound **536** has certain α-glucosidase inhibitory activity, with an IC_50_ of 377.42 ± 9.35 μmol/L [113]. Compounds **538** and **547** are natural steroid compounds, distributed in various plants of *Dendrobium*; numerous studies have shown that β-sitosterol has pharmacological activities such as antibacterial, anti-inflammatory, anti-tumor and anti-hyperlipidemia activity [157], and carotene has pharmacological effects such as inhibiting oxidative stress [158] and anti-tumor, antioxidant and neuroprotective effects [159], but currently there are still few studies on the activity of β-sitosterol and carotene in the genus *Dendrobium*.

### 2.12. Nucleosides

**Figure 12:**
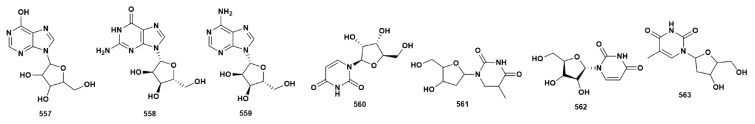
Structure of nucleoside compounds in *Dendrobium*.

Currently, seven nucleosides have been isolated from nine species of *Dendrobium* (**557**–**563**) [32,37,39,48,63,108,132,137,160,161]. The chemical structures of these nucleosides are shown in Figure 12, while Table S1 provides the source information for each compound. Nucleoside compounds are important metabolic products in process-of-life activities and have various effects such as anti-tumor, antiviral, immune regulation, and antibacterial and anti-inflammatory effects [162]. Liu et al. found that the content of nucleoside compounds varies significantly among different species of the genus *Dendrobium*. For example, the content of guanosine and adenosine in *D. officinale* is relatively abundant [163]. Wu et al. established a high-performance liquid chromatography method for the determination of nucleoside components (guanosine, adenosine, and uridine) in Guangxi *D. officinale*, and determined the content of nucleoside components with different growth years and planting patterns, providing a certain basis for the quality evaluation of *D. officinale* [164]. However, current research on the nucleoside activity of the genus *Dendrobium* is still relatively scarce, which may be related to its relatively low distribution in *Dendrobium*.

### 2.13. Fluorenone Compounds

**Figure 13:**
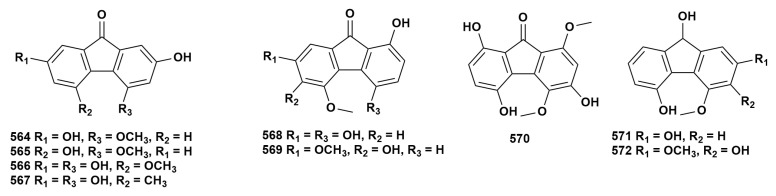
Structure of fluorenone compounds in *Dendrobium*.

At present, nine fluorenones (**564**–**572**) have been isolated from 12 species of *Dendrobium* [16,43,61,69,73,75,83,88,91,92,124,125,131,133,143,144]. Fluorenones are characterized by condensed aromatic rings and carbonyl functional groups, typically featuring three to five hydroxyl or methoxyl substitutions. The chemical structure of these compounds is depicted in Figure 13, while Table S1 provides detailed source information. The furanone compounds in the genus *Dendrobium* possess pharmacological activities such as neuroprotection and anti-tumor effects. Zhang et al. [144] demonstrated that compound **564** has inhibitory activity against acetylcholinesterase. Compounds **566** and **568** have significant inhibitory effects on the growth of human liver cancer BEL-7402 cells, with IC_50_ values of 1.38 μg/mL and 0.97 μg/mL respectively [12].

### 2.14. Others

**Figure 14:**
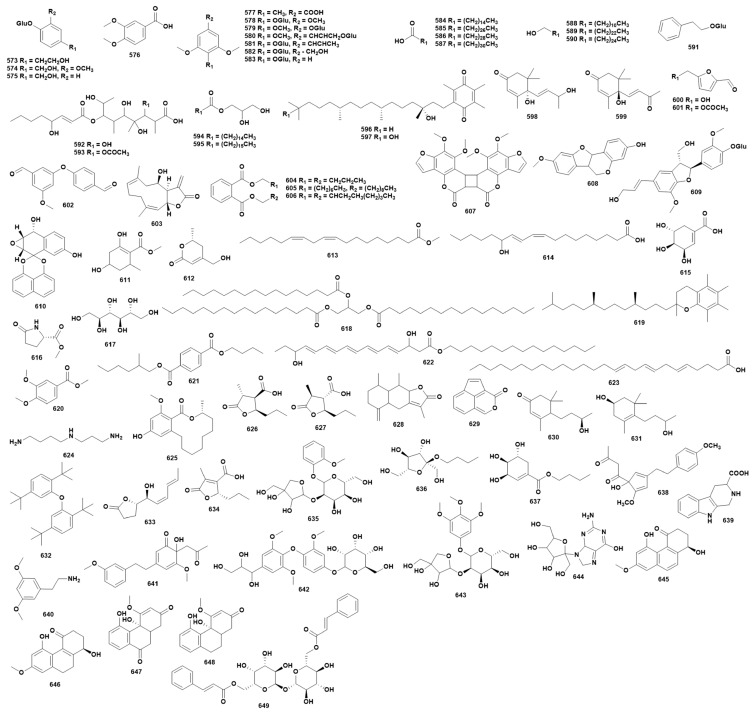
Structure of other compounds in *Dendrobium*.

In addition to fatty acids and small molecule lactones, various other compounds have been isolated from species of the genus *Dendrobium*. Currently, 77 additional compounds (**573**–**649**) have been identified from 21 different *Dendrobium* species [16,25,27,29,37,39,41,42,48,52,68,74,85,86,87,94,99,109,112,115,117,118,121,128,131,132,133,134,135,136,137,144,154,165,166,167]. The chemical structures of these compounds are depicted in Figure 14, while Table S1 provides detailed source information. The pharmacological activities of other types of compounds isolated from the genus *Dendrobium* have been less studied. Zhao et al. [168] found that compound **574** can stimulate B cell proliferation and inhibit T cell proliferation in vitro. Zhang et al. [169] found that compound **577** showed stronger activity in the oxygen free radical scavenging ability test than the antioxidant vitamin C.

### 2.15. Summary of Chemical Diversity and Active Components

In summary, the genus *Dendrobium* exhibits extremely high chemodiversity, with more than 640 metabolites isolated and identified to date. Among them, *D. officinale*, *D. nobile*, *D. huoshanense*, *D. chrysotoxum*, and *D. fimbriatum*, which are included in the Chinese Pharmacopoeia, are the most “chemically rich” species, with more than 100 metabolites isolated from each species, and are also the most widely studied species in pharmacological research.

From the perspective of chemical classification, these identified metabolites can be divided into 14 major categories according to their core structural skeletons, with significant differences in quantity, species distribution and biological activity across categories. The most abundant category is phenolic compounds, with 121 isolates obtained from 29 *Dendrobium* species, followed by bibenzyls (102 compounds from 32 species), which together form the main body of secondary metabolites in this genus. Phenolic compounds show extensive structural diversity and a wide range of pharmacological activities such as antioxidant, antibacterial and anti-tumor effects; bibenzyls are widely distributed in the genus *Dendrobium* and have been a research hotspot for their excellent anti-inflammatory, antioxidant and anti-tumor activities. Other major categories with abundant structural diversity include flavonoids (76 compounds), phenanthrene compounds (65 compounds), terpenoids (53 compounds), and alkaloids (49 compounds divided into seven subclasses), among which alkaloids, as the signature bioactive constituents of the genus *Dendrobium*, have been well documented for their cardiovascular protective effects including lipid regulation, anti-inflammation and myocardial protection. The rich and diverse structural types of these metabolites not only fully demonstrate the chemodiversity of the *Dendrobium* genus, but also provide the core material basis for its multiple pharmacological activities, especially the multi-target and multi-pathway cardiovascular protective effects elaborated on in this review.

The distribution of these metabolites shows significant interspecific differences across the *Dendrobium* genus. The complete species source information of all compounds is detailed in Table S1 of the Supplementary Materials, which fully demonstrates the chemodiversity of the entire genus, and provides the material basis for the differences in pharmacological activities among different *Dendrobium* species.

## 3. Application and Pharmacological Mechanism of Active Components of Dendrobium in CVDs

**Figure 15:**
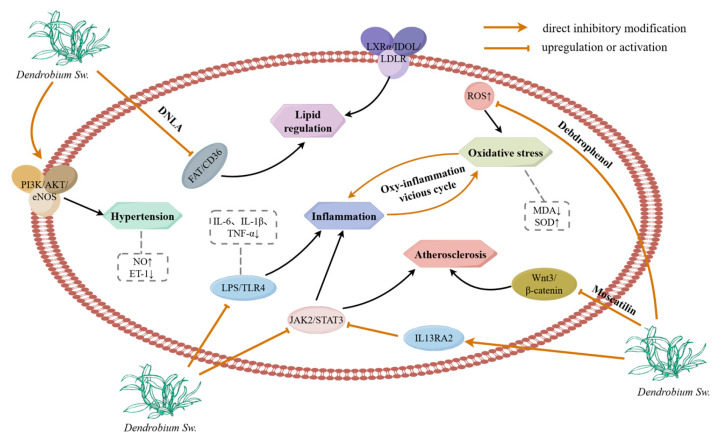
The pharmacological mechanism of *Dendrobium* in preventing CVDs. Note: This figure was drawn by Figdraw.

A large amount of evidence indicates that extracts of the genus *Dendrobium* and its active metabolites can exert cardiovascular protection through multiple targets and pathways. The core mechanism involves effectively inhibiting oxidative stress, regulating lipid metabolism disorders, reducing inflammatory responses, delaying the progression of atherosclerosis, inhibiting myocardial fibrosis, as well as exerting significant anti-hypertensive effects and improving vascular endothelial dysfunction, which cover the key pathological links of the whole process of CVDs (Figure 15).

### 3.1. Antioxidant Stress

The antioxidant stress-mediated cardiovascular protective effects have been reported in multiple species of the *Dendrobium* genus, including *D. officinale*, *D. nobile*, *D. pachyglossum* and other species. Oxidative stress is characterized by excessive production of reactive oxygen species (ROS) and impaired antioxidant defense systems, and it is the core initiating link of cardiovascular pathological damage. It can directly induce cardiomyocyte apoptosis, vascular endothelial cell damage, and lipid peroxidation, and further promote the occurrence and development of atherosclerosis, myocardial fibrosis and other CVDs [170]. A large number of studies have shown that extracts and active metabolites of the genus *Dendrobium* can effectively alleviate cardiovascular oxidative stress damage through multi-dimensional antioxidant regulation. Zhang et al. [171] found that the extract of *D. officinale* could reduce the malondialdehyde (MDA) level in myocardial tissue by 35% and increase the superoxide dismutase (SOD) activity by 41% in a streptozotocin-induced diabetic cardiomyopathy mouse model, effectively inhibiting oxidative stress-mediated myocardial damage. Han et al. [172] demonstrated that the water extract of *D. officinale* could significantly increase the SOD activity in zebrafish with atherosclerosis and reduce their MDA content.

In terms of monomer compounds, organic amine-type alkaloids (**44**) from *D*. *devonianum* show a good free radical scavenging ability with an IC_50_ of 1.61 mmol/L [46]; bibenzyl compound **132** exhibits a significant antioxidant protective effect in keratinocytes against H_2_O_2_-induced oxidative stress [72]; and terpenoid compound **531** also has strong antioxidant activity with an IC_50_ of 363.77 ± 3 μM for DPPH free radical scavenging [27]. These active compounds constitute the core material basis for the antioxidant stress effect of species of the genus *Dendrobium* in the cardiovascular system.

### 3.2. Lipid Regulation

The lipid-regulating activity related to cardiovascular protection has been mainly confirmed in *D. nobile*. Lipid metabolism disorders are characterized by elevated low-density lipoprotein cholesterol (LDL-C), increased triglycerides (TG), and decreased high-density lipoprotein cholesterol (HDL-C), which are major risk factors for atherosclerosis and coronary heart disease [173]. Studies have shown that plants of the genus *Dendrobium* and their active metabolites can comprehensively regulate lipid metabolism through multiple pathways, such as inhibiting lipid synthesis, promoting lipid breakdown and uptake, and reducing ectopic lipid deposition, thereby alleviating cardiovascular damage caused by abnormal blood lipids. Pan et al. [174] found that *D. nobile* Lindl. alkaloids (DNLAs) can inhibit the abnormal expression of fatty acid translocase enzyme (FAT/CD36) in myocardial tissue, reduce the rate of free fatty acid uptake in the myocardial ischemia–reperfusion injury model, and decrease the abnormal accumulation of LCACoA in myocardial tissue, thereby improving myocardial energy metabolism disorder. The alkaloids of *D. nobile* can also improve lipid metabolism disorder by regulating the LXRα/IDOL/LDLR pathway, increase the uptake of the 1,1′-dioctadecyl-3,3,3′,3′-tetramethyl-indocarbocyanideperchlorate low-density lipoprotein (LDL) by HepG2 cells to alleviate the effects caused by LPS, and thereby improve lipid metabolism disorder [175].

### 3.3. Anti-Atherosclerosis

The anti-atherosclerotic effects have been confirmed in a variety of *Dendrobium* species, mainly *D. huoshanense* and *D. officinale*. Atherosclerosis is the pathological basis of most acute cardiovascular events. Its pathological process involves endothelial dysfunction of blood vessels, phenotypic transformation of vascular smooth muscle cells, infiltration of inflammatory cells, vascular calcification, and formation of lipid plaques [176,177]. The plants of the genus *Dendrobium* and their active metabolites can intervene in the entire process of atherosclerosis through multi-target regulation and delay the progression of atherosclerotic lesions. Vascular calcification is a devastating vascular complication of atherosclerotic cardiovascular diseases and chronic kidney diseases, which increases the incidence of adverse cardiovascular events and reduces the efficacy of vascular interventional therapy. Zhang et al. [178] found that Moscatilin in *D. huoshanense* could bind to the IL13RA2 subunit of interleukin 13 receptor and inhibit the signal transducer and activator of transcription 3 (STAT3) and Wnt3/β-catenin pathways, thereby reducing vascular smooth muscle cell calcification. Fan et al. [179] identified six main active components from the water extract of *D. huoshanense*, among which Dendrophenol could increase the NO activity of low shear stress (LSS)-induced endothelial cells in the atherosclerotic zebrafish model and reduce the ROS level. Li et al. [180] identified MMP9, CCR1 and HMOX1 as the key mediators of nicotine exposure-induced atherosclerosis through multi-dimensional artificial intelligence analysis, combined with in vitro experiments and simulation verification analysis. These targets can be effectively inhibited by the active components of *D. officinale*, such as erianin, nobilin D, naringenin, dihydroquercetin, citric acid, eugenol, vanillic acid and L-tryptophan.

### 3.4. Anti-Inflammatory and Anti-Myocardial Fibrosis

Multiple species of the *Dendrobium* genus, including *D. officinale*, *D. nobile*, *D. huoshanense* and *D. crepidatum,* have been reported to exert cardiovascular anti-inflammatory and anti-myocardial fibrosis effects. Chronic inflammatory response is the core pathological mechanism throughout the entire process of cardiovascular diseases. Persistent inflammatory stimulation will further promote the activation of myocardial fibroblasts, excessive deposition of the extracellular matrix, and myocardial fibrosis, ultimately leading to cardiac remodeling and heart failure [181,182]. The plants of the genus *Dendrobium* and their active metabolites can effectively alleviate cardiovascular inflammatory damage and inhibit the progression of myocardial fibrosis by inhibiting key inflammatory signaling pathways and reducing the secretion of pro-inflammatory factors. The extract of *D. officinale* can inhibit the LPS/TLR4 pathway and reduce the levels of inflammatory mediators such as IL-6 and TNF-α in the serum of rats with metabolic hypertension [183]. Liu et al. [184] found that the *D. nobile* Lindl. alkaloids (DNLAs) can alleviate the inflammatory response and cell apoptosis in myocardial ischemia–reperfusion injury by inhibiting the JAK2/STAT3 pathway.

In terms of monomer compounds, bibenzyl compounds **52** and **53** from *D. huoshanense* show significant anti-inflammatory effects, which can inhibit NO release from LPS-induced RAW264.7 cells in a concentration-dependent manner; at 80 μmol/L, the inhibition rates of TNF-α mRNA expression are 38.4% and 39.4%, and the inhibition rates of IL-1β mRNA expression are 65.6% and 58.9%, respectively [55]. Indolizidine alkaloids (**35**) and piperidine alkaloids (**37**) also exhibit strong inhibitory effects on NO release [36], which are important active substances for the anti-inflammatory effect of the genus *Dendrobium* in the cardiovascular system.

### 3.5. Anti-Hypertensive Effect and Vascular Endothelial Dysfunction Improvement

The anti-hypertensive and vascular endothelial protective effects have been observed in *D. officinale* and other medicinal species of the *Dendrobium* genus. Hypertension is one of the most important independent risk factors for cardiovascular diseases, and vascular endothelial dysfunction is the core early pathological change in hypertension and its target organ damage [185]. Normal vascular endothelial function depends on the balance between endothelium-derived vasodilatory factors (such as NO) and vasoconstrictive factors (such as endothelin-1). Impaired synthesis and bioavailability of endothelin, as well as excessive activation of endothelin-1, can lead to increased vascular tension, abnormal vascular contraction, and elevated blood pressure, and further promote the occurrence and development of cardiovascular complications such as atherosclerosis and myocardial hypertrophy [186].

A growing number of studies have confirmed that the genus *Dendrobium* and its active metabolites exert significant anti-hypertensive effects and vascular endothelial protective effects. Yin et al. [187] discovered that the compound of the genus *Dendrobium* can reduce the collagen deposition in the aorta of hypertensive rats and alleviate myocardial fibrosis, and this intervention also plays a prominent antihypertensive role by regulating lipid metabolism, balancing serum vascular endothelial function-related indicators, activating the PI3K/AKT/eNOS signaling pathway, improving thoracic aortic pathological changes, and inhibiting excessive expression of adhesion factors, thereby effectively improving multiple cardiovascular lesions induced by hypertension. Li et al. [188] discovered that *D. officinale* can effectively lower the blood pressure of rats with metabolic hypertension caused by dietary habits and improve lipid disorders by regulating the structure of the intestinal microbiota, increasing the level of short-chain fatty acids, activating the intestinal–vascular axis SCFA-GPCR43/41 signaling pathway, up-regulating eNOS expression and NO production, and restoring vascular endothelial relaxation function.

The complete originating species, source and reference information for all active compounds mentioned in this section are detailed in Table S1 of the Supplementary Materials.

## 4. Challenges and Prospects for Clinical Application

### 4.1. Limitations of This Review

While this review has systematically assessed the research progress on the chemical constituents and cardiovascular protective effects of the genus *Dendrobium*, it is necessary to clarify the inherent limitations of the current work and the existing research gaps in this field. At present, the research on the cardiovascular protective effects of plants in the genus *Dendrobium* mainly focuses on in vitro cell experiments and animal model studies, while clinical evaluations on humans are extremely limited. The existing clinical studies have the following obvious deficiencies: The scale and quality of clinical trials are insufficient. Most related clinical studies are small-sample (sample size < 100 cases), single-center, non-randomized controlled observations, lacking large-scale, multi-center, randomized double-blind placebo-controlled trials with high-level evidence, which greatly limits the validity and clinical reference value of the existing evidence.

### 4.2. Challenges for Clinical Application

Beyond the limitations of the clinical trial scale and quality, there are still multiple core challenges hindering the clinical translation and application of *Dendrobium* in the cardiovascular field. First, the selection of clinical outcome indicators is not rigorous enough. Most existing studies use blood pressure, blood lipids, inflammatory factors, etc., as secondary evaluation indicators, while lacking follow-up data on hard clinical endpoints such as major adverse cardiovascular events (MACEs), all-cause mortality, and cardiovascular mortality. Second, the dose–effect relationship and long-term safety have not been clarified. There are no unified standards for the clinical intervention doses, administration routes, and treatment cycles of the active components and extracts of the genus *Dendrobium*, and no systematic assessment of the safety and potential adverse reactions of this drug for patients with cardiovascular diseases during long-term use. In addition, the pharmacokinetic characteristics and in vivo bioavailability of most monomer metabolites of *Dendrobium* in the human body are still unclear, which further limits its clinical translation and application.

## 5. Conclusions and Prospects

The genus *Dendrobium* as an important part of traditional Chinese medicine, and significant progress has been made in the research of chemical components and pharmacological activities in recent years. Studies have shown that the genus *Dendrobium* contains a variety of active components, including alkaloids, benzyl ethers, flavonoids, phenols, etc. These components exhibit clear pharmacological effects in anti-tumor, anti-inflammatory, antioxidant, hypoglycemic, and neuroprotective aspects. A large amount of evidence indicates that extracts of the genus *Dendrobium* and their active components can exert cardiovascular protection through multiple targets and pathways. The core mechanism involves effectively inhibiting oxidative stress, reducing inflammatory responses, regulating lipid metabolism disorders, delaying the progression of atherosclerosis, inhibiting myocardial fibrosis, lowering blood pressure, and improving vascular endothelial dysfunction, which are key pathological links.

In-depth research on the plants of the genus *Dendrobium* not only expands our scientific understanding of their traditional medicinal value, but also provides important clues and bases for the development of new drugs or functional products with cardiovascular protection effects. Currently, most studies still focus on the extract or mixed component level. The structure–activity relationship of specific monomeric compounds, the precise molecular mechanism of action, and standardized clinical efficacy evaluation remain the key directions that need to be explored in the future. In addition, the influence of different varieties, origins, and processing methods on the pharmacological substance basis also needs to be systematically clarified.

Overall, the genus *Dendrobium* has broad application prospects in the fields of medicine and health care. By further integrating modern technological means to clarify their pharmacological substances and mechanism of action, and promoting high-quality basic research that can be transformed into clinical practice, it is expected that new strategies and resources for the prevention and treatment of cardiovascular diseases will be provided.

## Data Availability

Not applicable.

## Supplementary Materials

The following supporting information can be downloaded at: https://www.mdpi.com/article/10.3390/ijms27094149/s1.
