# Supplementary material for: Chemical Constituents, Pharmacological Activities, and Cardiovascular Protective Mechanisms of Dendrobium Species: A Review
Source: Int J Mol Sci. 2026 May 6;27(9):4149. doi: 10.3390/ijms27094149 (PMC13164088; doi:10.3390/ijms27094149)
Supplement: Supplementary file 1 [file ijms-27-04149-s001.zip › ijms-4265897-supplementary.pdf]

Supplementary Material

Chemical Constituents, Pharmacological Activities, and Cardiovascular Protective Mechanisms of *Dendrobium* Species: A Review

Yue Hu [1], Zhiyong Li [1], Jian Li [1], Xiaowen Li [1,2] and Meina Wang [1,*]

[1] The Orchid Conservation & Research Center of Shenzhen and the National Orchid Conservation Center of China, Shenzhen Key Laboratory for Orchid Conservation and Utilization, Key Laboratory of National Forestry and Grassland Administration on Dendrobium Officinale, Shenzhen 518114, China; huyuea0914@126.com (Y.H.); lizhiyong83@hotmail.com (Z.L.); etecology@foxmail.com (J.L.); shelwin525@163.com (X.L.)

[2] School of Biological Sciences and Technology, Beijing Forestry University, Beijing 100083, China

* Correspondence: wangmn@cnocc.cn

Table S1 Compounds from the genus *Dendrobium.*

No.	Compounds name	Sources	References
**Alkaloids (1-49)**			
1	Dendrobine	*D. nobile*	[1]
2	(−)-(1*R*,2*S*,3*R*,4*S*,5*R*,6*S*,9*S*,11*R*)-11-carboxymethyldendrobine	*D. nobile*	[1]
3	Dendrine	*D. nobile*	[2]
4	Dendronobiline A	*D. nobile*	[3]
5	Dendramine	*D. nobile*	[4]
6	mubironine B	*D. nobile*	[1]
7	N-methyldendrobinium	*D. nobile*	[1]
8	N-isopentenyldendrobinium	*D. nobile*	[5]
9	Dendrobine-N-oxide	*D. nobile*	[5]
10	2-hydroxydendrobine	*D. findlayanum*	[6]
11	9-hydroxy-10-oxodendrobine	*D. nobile*	[7]
12	4-hydroxy-dendroxine	*D. nobile*	[8]
13	6-hydroxy-dendroxine	*D. nobile*	[9]
14	Dendroxine	*D. nobile*, *D. signatum*	[10,11]
15	N-isopentenyldendroxinium	*D. nobile*	[5]
16	N-isopentenyl-6-hydroxydendroxinium	*D. nobile*	[5]
17	Dendroterpene F	*D. nobile*	[12]

18	Dendrofindline B	*D. findlayanum*	[13]
19	Nobiline	*D. nobile*	[8]
20	Dendrofindline A	*D. findlayanum*	[13]
21	findlayine A	*D. findlayanum*	[13]
22	findlayine B	*D. findlayanum*	[6]
23	Dendroterpene A	*D. nobile*	[14]
24	7-hydroxydendroterpene	*D. signatum*	[10]
25	Dendroterpene B	*D. nobile*	[14]
26	Dendroterpene G	*D. nobile*	[12]
27	(−)-(1*R*,2*S*,3*R*,4*S*,5*R*,6*S*,9*S*,11*R*)-11-Carboxymethyldendrobine	*D. nobile*	[15]
28	(+)-(1*R*,2*S*,3*S*,4*R*,5*R*,6*S*,9*R*)-2,4,11-Trihydroxypicrotoxane-3(15)-lactone	*D. nobile*	[15]
29	Wardianumine A	*D. wardianum*	[16]
30	Hygrine	*D. polyanthum*, *D. chrysanthum*	[17,18]
31	findlayine C	*D. findlayanum*	[6]
32	(-)-dendroprimine	*D. polyanthum*, *D. chrysanthum*	[17,18]
33	Dendrocrepidine B	*D. crepidatum*	[19]
34	Dendrocrepidamine	*D. crepidatum*	[20]
35	(±)-homocrepidine A	*D. crepidatum*	[21]
36	crepidine	*D. crepidatum*	[21]
37	Homocrepidine B	*D. crepidatum*	[21]
38	periloyrine	*D. huoshanense*	[22]
39	Moniline	*D. moniliforme*	[23]
40	Niacinamide	*D. gratiosissimum*	[24]
41	N-*trans*-caffeoyltyramine	*D. devonianum*	[25]
42	Dihydro-feruloyltyramine	*D. officinale*	[26]
43	N-*trans*-cinnamoyl tyramine	*D. nobile*, *D. gratiosissimum*, *D. wardianum*, *D. officinale*, *D. hercoglossum*	[24,27-30]
44	N-*trans*-feruloyltyramine	*D. polyanthum*, *D. devonianum*	[17,31]
45	N-*trans*-feruloyl tyramine	*D. nobile*	[30]

46	N-*trans*-*p*-coumaroyl tyramine	*D. nobile*, *D. polyanthum*, *D. devonianum*, *D. officinale*	[17,30-32]
47	N-*cis*-*p*-coumaroyl tyramine	*D. nobile*, *D. devonianum*	[25,30]
48	N-*cis*-feruloyl tyramine	*D. nobile*, *D. officinale*	[26,30]
49	Caffeine	*D. aphyllum*	[33]
**Bibenzyls (50-151)**			
50	4,4'-dihydroxybibenzyl	*D. officinale*	[34]
51	3-hydroxy-5-methoxybibenzyl	*D. nobile*, *D. moschatum*	[35,36]
52	3,4'-Dihydroxy-5-Methoxybibenzyl	*D. officinale*, *D. crystallinum*, *D. heterocarpum*, *D. huosha nense*, *D. wardianum*, *D. grat iosissimum*, *D. findlayanum*	[13,29,37-41]
53	Batatasin III	*D. officinale*, *D. strongylanthu m*, *D. loddigesii*, *D. crystallin um*, *D. nobile*, *D. heterocarpu m*, *D. huoshanense*, *D. aphyll um*, *D. moschatum*, *D. bellatu lum*, *D. chrysotoxum*, *D. poly anthum*	[26,30,36,39,40,42-48]
54	3-hydroxy-4',5-dimethoxybibenzyl	*D. officinale*, *D. heterocarpu m*, *D. huoshanense*, *D. devoni anum*	[34,40,49,50]
55	Dihydroresveratrol	*D. officinale*, *D. crystallinum*, *D. huoshanense*, *D. aphyllum*	[41,44,51,52]
56	3,3',5-trihydroxybibenzyl	*D. loddigesii*, *D. nobile*	[35,46]
57	3-Methoxystrobinol	*D. nobile*, *D. heterocarpum*, *D. bellatulum*, *D. chrysotoxum*	[1,40,42,45]
58	gigantol	*D. moniliforme*, *D. nobile*, *D. heterocarpum*, *D. huoshanens e*, *D. aphyllum*, *D. wardianu m*, *D. chrysotoxum*, *D. crystal linum*, *D. denneanum*, *D. trig onopus*, *D. pachyglossum*, *D. palpebrae*, *D. polyanthum*, *D. officinale*	[16,38,40,41,43-45,52-58]
59	3',4-dihydroxy-3,5'-dimethoxybibenzyl	*D. nobile*, *D. huoshanense*, *D. hancockii*	[35,39,59]
60	4,5′-dihydroxy-3,5-dimethoxybibenzyl	*D. ellipsophyllum*	[60]
61	tristin	*D. officinale*, *D. loddigesii*, *D. nobile*, *D. huoshanense*, *D. trigonopus*, *D. devonianum*,	[1,26,43-45,49,52,53,61]

		D. aphyllum, *D. chrysotoxum*, *D. polyanthum*	
62	gigantol tetramethyl ether	*D. gratiosissimum*	[37]
63	3,5,3'-trimethoxy-4'-hydroxybibenzyl	*D. crystallinum*, *D. wardianu m*, *D. gratiosissimum*	[37,47,62]
64	4,4'-dihydroxy-3,5-dimethoxybibenzyl	*D. officinale*, *D. crystallinum*, *D. gratiosissimum*, *D. findlay anum*, *D. signatum*, *D. willia msonii*, *D. huoshanense*, *D. a phyllum*, *D. loddigesii*	[10,13,22,3 7,38,46,63-65]
65	3,4,4′-trihydroxy-5-methoxybibenzyl	*D. findlayanum*	[13]
66	dendrosinen B	*D. huoshanense*	[52]
67	3,4-dihydroxy-4',5-dimethoxy bibenzyl	*D. officinale*, *D. heterocarpu m*, *D. huoshanense*, *D. gratio sissimum*, *D. wardianum*, *D. moniliforme*	[16,37,38,4 0,52,66]
68	4,5-dihydroxy-3,5′-dimethoxybibenzyl	*D. nobile*	[1]
69	3,4,4'-trihydroxyl-5,3'-dimethoxybenzyl	*D. gratiosissimum*, *D. pachygl ossum*, *D. palpebrae*	[37,56,57]
70	Dendrocandin E	*D. officinale*	[51]
71	3,4-dihydroxyl-5,3',4'-trimethoxybibenz yl	*D. gratiosissimum*, *D. findlaya num*	[13,37]
72	cumulatin	*D. crepidatum*	[60]
73	3,3′-Dihydroxy-4,5-Dimethoxybibenzyl	*D. officinale*, *D. bellatulum*	[38,42]
74	3,4′-Dihydroxy-4,5-Dimethoxybibenzyl	*D. officinale*	[38]
75	dendrogratiol A	*D. gratiosissimum*	[37]
76	DTB	*D. gratiosissimum*	[37]
77	4′-Hydroxy-3′,5′-dimethoxybibenzyl	*D. officinale*	[26]
78	4,5,4′-trihydroxy-3,3′-dimethoxybibenz yl	*D. ellipsophyllum*	[60]
79	aloifol I	*D. hercoglossum*, *D. bellatulu m*, *D. findlayanum*, *D. willia msonii*, *D. gibsonii*	[13,42,65,6 7,68]
80	4-hydroxy-3,5′,5-trimethoxybibenzyl	*D. nobile*	[1]
81	Amoenylin	*D. wardianum*, *D. gratiosissim um*	[37,62]
82	4-[2-(3-hydroxy-4-methoxyphenyl)ethy l]-2,6-dimethoxyphenol	*D. chrysotoxum*	[45]
83	chrysotoxin	*D. officinale*, *D. fimbriatum*,	[26,37,45,5

		D. nobile, *D. sinense*, *D. den neanum*, *D. gratiosissimum*, *D. chrysotoxum*	5,69-71]
84	Moscatilin	*D. hercoglossum*, *D. nobile*, *D. officinale*, *D. huoshanense*, *D. findlayanum*, *D. hancocki i*, *D. chrysanthum*, *D. thyrsifl orum*, *D. loddigesii*, *D. crepid atum*, *D. fimbriatum*, *D. heter ocarpum*, *D. aphyllum*, *D. de nneanum*, *D. trigonopus*, *D. st uposum*, *D. moschatum*, *D. gr atiosissimum*, *D. wardianum*, *D. chrysotoxum*, *D. pachyglos sum*, *D. williamsonii*, *D. palp ebrae*, *D. polyanthum*	[13,16,20,2 8,36-38,40, 43,52-54,5 6,57,59,63,6 5,71-77]
85	chrysotobibenzyl	*D. officinale*, *D. chrysanthum*, *D. fimbriatum*, *D. nobile*, *D. huoshanense*, *D. denneanum*, *D. chrysotoxum*	[26,52,55,6 9,76,78,79]
86	isoamoenylin	*D. chrysotoxum*	[45]
87	crepidatin	*D. chrysanthum*, *D. crepidatu m*, *D. nobile*, *D. chrysotoxum*	[20,45,55,7 6]
88	Erianin	*D. officinale*, *D. huoshanense*, *D. chrysotoxum*, *D. nobile*	[26,45,50,8 0]
89	3,4-dihydroxyl-5,4'-dimethoxystilbene	*D. gratiosissimum*	[37]
90	4-hydroxy-3,5,4'-trimethoxystilbene	*D. gratiosissimum*	[37]
91	4-hydroxy-3,5,3'-trimethoxystilbene	*D. nobile*	[1]
92	oxyresveratrol	*D. huoshanense*	[50]
93	Dendrocandin A	*D. officinale*, *D. huoshanense*, *D. wardianum*, *D. heterocarp um*	[16,40,51,5 2]
94	Dendrocandin C	*D. officinale*, *D. huoshanense*	[51,52]
95	Nobilin B	*D. nobile*, *D. crepidatum*	[1,20]
96	Nobilin C	*D. nobile*, *D. chrysotoxum*	[1,45]
97	4,5-dihydroxy-3,3',α-trimethoxybibenzy l	*D. hercoglossum*, *D. nobile*	[28,74]
98	(*S*)-3,4,*α*-Trihydroxy-5,4′-dimethoxybib enzyl	*D. officinale*	[38]
99	3,4,α-trihydroxy-5,3′-dimethoxybibenzy l	*D. hercoglossum*	[28]

100	dendrohanol A	*D. hancockii*	[59]
101	4,α-dihydroxy-3,5,3'-trimethoxybibenzyl	*D. hercoglossum*, *D. nobile*	[28,74]
102	Nobilin D	*D. nobile*	[55]
103	aphyllals D	*D. aphyllum*	[44]
104	aphyllals E	*D. aphyllum*	[44]
105	(*R*)-3,*α*-dihydroxy-4,4',5-trimethoxybibenzyl	*D. findlayanum*	[13]
106	(S)-3,4,*α*-trihydroxy-4',5-dimethoxybibenzyl	*D. heterocarpum*	[40]
107	2,2′,5,5′-Tetramethyl-1,1′-bibenzyl	*D. officinale*	[26]
108	Dendrocandin D	*D. officinale*	[51]
109	Dendromoniliside E	*D. officinale*	[51]
110	Dendrocandin H	*D. officinale*	[51]
111	Dendrocandin L	*D. officinale*	[26]
112	Dendrocandin B	*D. huoshanense*, *D. wardianum*, *D. signatum*, *D. officinale*, *D. moniliforme*	[10,29,38,50,58]
113	Dendronbiline C	*D. nobile*	[81]
114	Dendrocandin U	*D. huoshanense*, *D. wardianum*, *D. officinale*	[16,38,52]
115	Dendrocandin T	*D. hercoglossum*, *D. huoshanense*	[52,67]
116	Dendronbiline B	*D. nobile*	[81]
117	Dendrocandin V	*D. officinale*, *D. wardianum*, *D. nobile*	[1,16,82]
118	Dendrocandin W	*D. officinale*	[82]
119	Dendrocandin O	*D. officinale*	[26]
120	Dendrocandin P	*D. officinale*	[26]
121	Dendrocandin Q	*D. officinale*	[26]
122	Dendronbibisline D	*D. nobile*	[83]
123	Dendrocandin I	*D. officinale*, *D. heterocarpum*, *D. signatum*	[10,40,51]
124	Didendronbiline A	*D. nobile*	[81]
125	Dendrocandin F	*D. officinale*, *D. heterocarpum*	[40,51]
126	Dendrocandin K	*D. officinale*	[26]

127	Dendrocandin G	*D. officinale*	[51]
128	dendrosinen C	*D. sinense*	[84]
129	Dendrocandin J	*D. officinale*	[26]
130	Nobilin E	*D. nobile*	[55]
131	dendrosinen D	*D. bellatulum*	[42]
132	dendropachol	*D. pachyglossum*	[56]
133	Denchrysotonol A	*D. chrysotoxum*	[45]
134	Denchrysotonol B	*D. chrysotoxum*	[45]
135	trigonopol B	*D. loddigesii*, *D. aphyllum*, *D. trigonopus*	[44,46,85]
136	Trigonpol B	*D. officinale*	[26]
137	3-(2-(7-methoxybenzo[1,3] dioxol-5-yl) ethyl)phenol	*D. williamsonii*	[65]
138	aphyllals C	*D. aphyllum*	[44]
139	densiflorol A	*D. officinale*, *D. heterocarpum*	[40,86]
140	dendrowillol A	*D. wardianum*, *D. williamsoni i*	[29,65]
141	6''-de-O-methyldendrofindlaphenol A	*D. signatum*, *D. officinale*	[10,38]
142	dendronbibisline C	*D. nobile*	[83]
143	crepidatuol B	*D. hancockii*	[59]
144	Dendrocandin N	*D. huoshanense*	[52]
145	Dendrocandin M	*D. officinale*	[26]
146	dendronophenol A	*D. nobile*	[1]
147	dendronophenol B	*D. nobile*	[1]
148	dendrofindlaphenol B	*D. officinale*	[82]
149	Denofficin	*D. officinale*	[26]
150	Dendrocandin X	*D. officinale*	[87]
151	trigonopols A	*D. trigonopus*	[85]
**Flavonoids (152-227)**			
152	Apigenin	*D. officinale*, *D. fimbriatum*, *D. gratiosissimum*	[26,88,89]
153	Chrysoeriol	*D. officinale*, *D. thyrsiflorum*	[26,77]
154	Tangeretin	*D. officinale*	[26]
155	Apigenin-6,8-di-C-*β*-*D*-glucopyranose	*D. officinale*, *D. huoshanense*	[26,52]

156	Apigenin-6,8-di-C-*α*-*L*-arabinopyranose	*D. huoshanense*, *D. fimbriatum*	[52,88]
157	Apigenin-6-C-*α*-*L*-arabinose 8-C-*β*-*D*-xylose	*D. officinale*	[26]
158	Apigenin-6-C-*β*-*D*-xylose-8-C-*α*-*L*-arabinose	*D. officinale*	[26]
159	Apigenin-6-C-*α*-*L*-arabinoid-(1→2)-O-*β*-*D*-glucose	*D. officinale*	[26]
160	Apigenin-8-C-glucose-(1→2)-*α*-*L*-arabinose	*D. officinale*	[26]
161	Apigenin-8-C-*α*-*L*-arabinofuranoside	*D. officinale*	[26]
162	Violanthin	*D. officinale*, *D. fimbriatum*	[88,90]
163	Apigenin-6-C-*α*-*L*-rhamnopyranosyl-8-C-*β*-*D*-xylopyranoside	*D. officinale*	[26]
164	Apigenin-6-C-*β*-*D*-xylopyranosyl-8-C-*α*-*L*-rhamnopyranosyl	*D. officinale*	[26]
165	Schaftoside	*D. officinale*, *D. huoshanense*	[50,90]
166	Isoschaftoside	*D. officinale*, *D. huoshanense*	[50,90]
167	Apigenin-6-C-*β*-*D*-xylose-8-C-*β*-*D*-glucopyranose	*D. officinale*	[26]
168	Apigenin-6-C-*β*-*D*-glucopyranose-8-C-*β*-*D*-xylose	*D. officinale*	[26]
169	Apigenin 7-glucoside	*D. officinale*	[26]
170	apigenin-7-O-*β*-*D*-glucopyranoside	*D. officinale*, *D. devonianum*	[49,91]
171	Isoviolanthin	*D. officinale*, *D. fimbriatum*	[88,90]
172	Vitexin-2″-O-*β*-*D*-glucopyran oside	*D. officinale*	[26]
173	vitexin-2″-O-rhamnoside	*D. huoshanense*	[50]
174	isovitexin	*D. fimbriatum*, *D. huoshanense*	[50,88]
175	Kaempferol	*D. officinale*, *D. crystallinum*	[26,47]
176	Quercetin	*D. officinale*	[26]
177	Isorhamnetin	*D. officinale*	[26]
178	Rutin	*D. officinale*, *D. huoshanense*, *D. devonianum*	[38,49,50]
179	Isoquercitrin	*D. officinale*	[26]
180	Hyperoside	*D. officinale*	[26]
181	Isorhamnetin-3-O-*β*-*D*-glucose	*D. officinale*	[26]
182	Isorhamnetin-3-O-*α*-*L*-rhamnose-(1→2)	*D. officinale*	[92]

	-*β*-*D*-glucopyranose		
183	Kaempferol-3-O-rutinoside	*D. officinale*	[26]
184	Astragalin	*D. officinale*	[26]
185	5,7,4′-trihydroxy-3′,5′-dimethoxy dihydroflavone	*D. wardianum*	[62]
186	apigenin-6-C-*α*-*L*-rhamnoside	*D. fimbriatum*	[88]
187	apigenin-7-O-*L*-rhamnopyranosyl-(1→6)-1-*β*-*D*-glucopyranoside	*D. devonianum*	[49]
188	apigenin-6-C-*α*-*L*-rhamnosyl-8-C-*β*-*D*-quinovoside	*D. officinale*, *D. fimbriatum*	[88,90]
189	Tricin	*D. aphyllum*, *D. wardianum*	[62,93]
190	4′-methoxyl-tricin	*D. aphyllum*	[93]
191	7,3',5'-tri-O-methyl-tricetin	*D. aphyllum*	[93]
192	chryseriol-7-O-*β*-*D*-glucoside	*D. fimbriatum*	[88]
193	Isoliquiritigenin	*D. officinale*	[94]
194	Naringenin chalcone	*D. officinale*	[26]
195	2′,3,4,4′,6′-Pentahydroxy chalcone	*D. officinale*	[26]
196	4,2′,4′,6′-Tetrahydroxy-3-methoxy-chalcone	*D. officinale*	[26]
197	Dihydrotricetin	*D. officinale*	[26]
198	Eriodictyol	*D. officinale*	[26]
199	Homoeriodictyol	*D. officinale*, *D. thyrsiflorum*, *D. chrysotoxum*	[26,45,77]
200	Hesperetin	*D. officinale*, *D. huoshanense*	[26,50]
201	Hesperidin	*D. officinale*, *D. polyanthum*	[17,94]
202	Liquiritigenin	*D. officinale*	[94]
203	3′,5,5′,7-tetrahydroxyflavanone	*D. officinale*	[95]
204	Naringenin	*D. thyrsiflorum*, *D. devonianum*, *D. gratiosissimum*, *D. trigonopus*, *D. wardianum*, *D. formosum*, *D. loddigesii*, *D. huoshanense*, *D. fimbriatum*, *D. crystallinum*, *D. nobile*, *D. officinale*	[1,24,29,46,49,50,53,64,77,94,96,97]
205	Naringenin-7-O-arabinoside	*D. officinale*	[26]
206	Taxifolin	*D. officinale*	[26]

207	tricin	*D. thyrsiflorum*	[77]
208	4',5,7-trihydroxy-6-methoxyflavanone	*D. crystallinum*	[41]
209	angophorol	*D. crystallinum*	[41]
210	blumeatin	*D. polyanthum*	[43]
211	5,4'-dihydroxy-7,3',5'-trimethoxyflavanone	*D. loddigesii*	[46]
212	balanophonin	*D. wardianum*	[29]
213	3',5'-*di*-C-Glucosylphloretin	*D. officinale*	[26]
214	Phlorizin	*D. officinale*	[26]
215	quercetin-3-O-*β-D*-glucopyranoside	*D. huoshanense*	[50]
216	baicalin	*D. huoshanense*	[50]
217	2′,4′-dihydroxy chalcone	*D. sinense*	[70]
218	biochanin A	*D. officinale*	[26]
219	carthamidin	*D. heterocarpum*	[40]
220	apigenin-6-C-*β-D*-glucoside-8-C-*β-D*-xyloside	*D. huoshanense*	[50]
221	Vitexin-2''-O-rhamnoside	*D. huoshanense*	[52]
222	6-C-(*α*-Arabinopyranosyl)-1-C-[(2-O-*α*-rhamnopyranosyl)-β-golactopyranosyl]-apigenin	*D. huoshanense*	[52]
223	3,5-dihydroxyflavone-7-O-glucuronopyranosid	*D. devonianum*	[49]
224	isorhamnetin-3-O-*β-D*-galactoside	*D. huoshanense*	[50]
225	isorhamnetin-3-O-*β-D*-glucopyranoside	*D. huoshanense*	[50]
226	quercetin-3-O-arabinoside	*D. huoshanense*	[50]
227	quercetin-3-O-glucoside	*D. huoshanense*	[50]
**Phenolic compounds (228-348)**			
228	4-Hydroxycinnamic acid	*D. officinale*, *D. wardianum*, *D. bellatulum*	[26,29,42]
229	trans-p-hydroxylethyl cinnamate	*D. sinense*	[70]
230	coniferyl aldehyde	*D. nobile*, *D. stuposum*, *D. d enneanum*	[1,72,98]
231	3-(4-Hydroxyphenyl)propionic acid	*D. officinale*, *D. formosum*, *D. nobile*, *D. devonianum*, *D. bellatulum*	[25,26,42,96,99]
232	*β*-Hydroxy propiovanillone	*D. officinale*	[26]

233	4-Hydroxy-3-methoxypropiophen one	*D. hercoglossum*, *D. officinale*	[26,67]
234	3-hydroxy-1-(4'-hydroxy-3',5'-dimethox yphenyl)-propan-1-one	*D. hercoglossum*, *D. wardianu m*, *D. williamsonii*	[29,65,67]
235	hydroxyphenylpropionic acid	*D. denneanum*	[100]
236	methyl melilotate	*D. moschatum*	[36]
237	ethyl melilotate	*D. moschatum*	[36]
238	caffeic acid	*D. bellatulum*	[42]
239	Ferulic Acid	*D. officinale*, *D. fimbriatum*, *D. aphyllum*, *D. bellatulum*	[26,42,97,1 01]
240	methyl 3-(*p*-hydroxyphenyl) propanoat e	*D. hercoglossum*, *D. huoshane nse*, *D. sinense*	[52,67,70]
241	Ethyl phydroxyphenylpropionate	*D. officinale*, *D. sinense*	[26,84]
242	Dihydroferulic acid	*D. huoshanense*	[52]
243	Dihydroconiferyl alcohol	*D. officinale*, *D. loddigesii*, *D. nobile*, *D. huoshanense*, *D. wardianum*	[26,29,52,1 02,103]
244	dihydroconifery acetate	*D. devonianum*	[25]
245	ethyl 4-hydroxycinnamate	*D. devonianum*	[25]
246	Coniferyl alcohol	*D. officinale*	[26]
247	scopoletin	*D. thyrsiflorum*, *D. chrysotoxu m*	[45,104]
248	Coniferyl *p*-coumarate	*D. officinale*, *D. formosum*	[26,96]
249	Sinapyl pcoumarate	*D. officinale*	[26]
250	dihydroconiferyl dihydro-*p*-coumarate	*D. strongylanthum*, *D. nobile*, *D. huoshanense*, *D. wardian um*, *D. bellatulum*	[1,22,29,42, 48]
251	4-Hydroxy-3-methoxypropiophen one	*D. officinale*	[26]
252	3-Hydroxy-4,5-dimethoxypropiophenon e	*D. officinale*	[26]
253	(*Z*)-4-Hydroxycinnamic acid	*D. officinale*	[26]
254	*eis*-*p*-hydroxyl ethyl cinnamate	*D. sinense*	[84]
255	*p*-Hydroxy-*trans*-cinnamic acid nonyl ester	*D. crystallinum*	[64]
256	*p*-Hydroxy-*trans*-cinnamic acid octaco syl ester	*D. crystallinum*	[64]
257	Tricyl *p*-hydroxy-trans-cinnamate	*D. officinale*, *D. chrysanthum*, *D. thyrsiflorum*, *D. aduncum*, *D. denneanum*, *D. nobile*, *D.*	[26,89,104-108]

		gratiosissimum	
258	*p*-Hydroxy-cis-cinnamic acid triaconty l ester	*D. officinale*, *D. aduncum*, *D. nobile*, *D. moniliforme*	[26,66,107, 109]
259	Tricyl phydroxycis cinnamate	*D. wardianum*	[29]
260	3-Methoxy-4-hydroxy-trans-cinnamic a cid tetracosyl ester	*D. crepidatum*, *D. nobile*	[109,110]
261	*trans*-Ferulic acid hexacosyl ester	*D. crystallinum*	[64]
262	Dioctadecyl transferulate	*D. officinale*, *D. fimbriatum*, *D. gratiosissimum*, *D. monilif orme*	[26,66,89,11 1]
263	Tinosporaic acid A	*D. officinale*	[26]
264	1-O-*p*-Feruloyl-*β*-*D*-glucopyranoside	*D. officinale*	[26]
265	Arillatose B	*D. officinale*	[26]
266	*p*-hydroxyphenylethyl-*p*-coumarate	*D. signatum*	[10]
267	*p*-hydroxyphenethyl *trans*-ferulate	*D. wardianum*	[29]
268	Shashenoside I	*D. officinale*	[26]
269	Michehedyoside C	*D. huoshanense*	[112]
270	4-Hydroxy-3,5-dimethoxy-*trans* cinna maldehyde	*D. officinale*	[26]
271	Chlorogenic acid	*D. officinale*	[26]
272	7-hydroxy-14-de-*O*-methyl-lasiodiplodi n	*D. formosum*	[96]
273	3,7,9-Trihydroxy-1-methyl benzo[c]chr omen-6-one	*D. officinale*	[26]
274	5,7'-dihydrochromone	*D. wardianum*	[29]
275	3′,4′,5′-trimethoxycinnamyl acetate	*D. officinale*, *D. nobile*	[1,26]
276	*p*-Hydroxy-cinnamic acid	*D. aphyllum*, *D. devonianum*	[25,33]
277	6-feruloyloxyhexanoic ester	*D. Sonia*	[113]
278	2-Hydroxycinnamic acid	*D. officinale*	[26]
279	2-methoxyphenol-5-hydroxymethyl	*D. loddigesii*	[103]
280	3-Hydroxy-4-methoxybenzyl alcohol	*D. nobile*	[1]
281	isovanillin	*D. loddigesii*	[103]
282	Syringaldehyde	*D. chrysanthum*, *D. nobile*, *D. sinense*, *D. aphyllum*	[1,33,84,11 4]
283	2′-hydroxypropiosyringone	*D. nobile*	[1]
284	tachioside	*D. denneanum*	[115]

285	*m*-hydroxybenzoic acid	*D. formosum*	[96]
286	3-hydroxybenzaldehyde	*D. sinense*	[84]
287	4-(6-hydroxyphenyl)-2′-butanone	*D. nobile*	[1]
288	Methyl 3,4-dihydroxybenzoate	*D. devonianum*	[49]
289	Protocatechuic acid	*D. hercoglossum*, *D. chrysotoxum*, *D. aphyllum*, *D. devonianum*	[25,28,33,116]
290	Syringic acid	*D. chrysanthum*, *D. nobile*, *D. huoshanense*, *D. aphyllum*, *D. wardianum*	[1,29,52,93,108]
291	*p*-Hydroxybenzyl alcohol	*D. crepidatum*	[117]
292	2-(4-hydroxyphenyl)-ethanol	*D. formosum*	[96]
293	*p*-Hydroxybenzoic acid	*D. formosum*, *D. crystallinum*, *D. huoshanense*, *D. aphyllum*, *D. devonianum*, *D. wardianum*, *D. williamsonii*	[25,29,33,52,64,65,96]
294	*p*-Hydroxybenzaldehyde	*D. chrysanthum*, *D. crepidatum*, *D. fimbriatum*, *D. nobile*, *D. aphyllum*, *D. wardianum*, *D. devonianum*, *D. gratiosissimum*	[1,29,33,49,89,97,110,114]
295	*p*-Hydroxyphenylacetic acid	*D. aphyllum*	[63]
296	2-(4-Hydroxyphenyl)ethyl-*β*-*D*-glucopyranoside salidrosid	*D. aphyllum*	[63]
297	*p*-coumarate tetradecyl	*D. nobile*	[1]
298	*p*-coumarate decyl	*D. nobile*	[1]
299	4-Hydroxy-3-methoxybenzyl alcohol	*D. hercoglossum*	[67]
300	4-hydroxy-3-methoxyphenylethanol	*D. loddigesii*	[103]
301	vanillin	*D. nobile*, *D. wardianum*, *D. williamsonii*	[1,29,65]
302	apocynin	*D. nobile*	[1]
303	Vanillic acid	*D. chrysanthum*, *D. aphyllum*, *D. chrysotoxum*	[33,114,116]
304	citrusin C	*D. devonianum*	[25]
305	docosyl ferulate	*D. nobile*, *D. wardianum*, *D. polyanthum*	[1,17,62]
306	1,3,5-trimethoxybenzene	*D. hercoglossum*	[67]
307	3,4,5-trimethoxyphenol	*D. hercoglossum*	[67]

308	leonuriside A	*D. officinale*, *D. devonianum*	[25,51]
309	3,5-Dimethoxy-4-hydroxyphenyl-1-O-*β*-*D*-glucoside	*D. officinale*, *D. nobile*	[1,26]
310	Salicylic acid	*D. williamsonii*	[65]
311	5-hydroxyphenylpropanol	*D. nobile*	[1]
312	4-Hydroxy-2,6-dimethoxyphenyl glucopyranoside	*D. officinale*	[26]
313	leonuriside C	*D. officinale*	[26]
314	2-hydroxy-4-methoxy-3,6-dimethylbenzoic acid	*D. bellatulum*	[42]
315	12'-hydroxy-*α*-tocopherol	*D. nobile*	[12]
316	7-aldehyde-*α*-tocopherol	*D. nobile*	[12]
317	meso-secoisolariciresinol	*D. hercoglossum*, *D. officinale*	[51,67]
318	(−)-secoisolariciresinol-4-O-*β*-*D*-glucopyranoside	*D. denneanum*	[54]
319	(7*S*,8*R*)-dehydrodiconiferyl alcohol-9′-β-glucopyranoside	*D. nobile*	[1]
320	5,7-dihydroxy-isobenzofuran	*D. formosum*	[96]
321	icariol A_2-4-O-*β*-*D*-glucopyranoside	*D. officinale*	[91]
322	4-hydroxy-2-methoxy-3,6-dimethylbenzoic acid	*D. heterocarpum*	[40]
323	(*R*)-3-methoxyl-1-(2,6-dihydroxy phenyl)-butan-1-one	*D. chrysotoxum*	[118]
324	(*R*)-2,3-dihydro-2,5-dihydroxy-2-methylchromen-4-one	*D. chrysotoxum*	[118]
325	(*E*)-4-(2-methoxyvinyl) benzene-1,2-diol	*D. nobile*	[1]
326	3,4-Dihydroxy-5-methoxybenzaldehyde	*D. huoshanense*	[52]
327	(2*R*,4*S*)-2,3-dihydro-2-methyl-benzopyran-4,5-diol	*D. chrysotoxum*	[118]
328	isoliquiritin	*D. nobile*	[1]
329	7-O-*α*-*D*-ribosyl-2,3-dihydro-5-hydroxy-2-methyl-chromen-4-one	*D. chrysotoxum*	[118]
330	7-O-*α*-*D*-ribosyl-5-hydroxy-2-methyl-4*H*-chromen-4-one	*D. chrysotoxum*	[118]
331	ozoroalide	*D. moschatum*	[36]
332	(+)-denobilone A	*D. nobile*	[1]
333	(−)-denobilone A	*D. nobile*	[1]

334	Dencoumarin	*D. Sonia*	[113]
335	Zhepiresinol	*D. nobile*	[1]
336	Glaberide I 4-O-*β*-*D*-glucopyranoside	*D. officinale*	[26]
337	3-hydroxymethyl-9 methoxy-2-(4'-hydroxy-3',5'-dimethoxyphenyl)-2,3,6,7-tetrahydrophenanthro [4,3-b] furan-5,11-diol	*D. bellatulum*	[42]
338	narcissin	*D. nobile*	[1]
339	4-(3′-Hydroxypropyl)-2,6-dimethoxyphenol-3′-O-*β*-*D*-glucoside	*D. officinale*	[26]
340	Dihydroconiferydihydro-*p*-cumarate	*D. devonianum*	[119]
341	*di*-[2-(4-hydroxyphenyl)] ethyl ether	*D. nobile*	[80]
342	neoolivil	*D. bellatulum*	[42]
343	Justiciresinol	*D. hercoglossum*	[67]
344	3,5'-dimethoxy-3',4,9'-trihydroxy-7',9-epoxy-8,8'-lignan	*D. loddigesii*	[103]
345	ficusal	*D. hercoglossum*	[67]
346	3-hydroxy-1-(4-hydroxy-3,5-dimethoxyphenyl)-2-[4-(3-hydroxy-1-(*E*)-propenyl)-2,6-dimethoxyphenoxy]propyl-7-O-*β*-*D*-glucopyranoside	*D. denneanum*	[120]
347	aphyllone B	*D. aphyllum*	[44]
348	Chromcinale	*D. officinale*	[26]
**Simple phenylpropanoids (349-361)**			
349	Benzene propanoic acid	*D. devonianum*	[119]
350	3-(3,4,5-trimethoxyphenyl)propyl acetate	*D. officinale*	[26]
351	Trans-3,4,5-Trimethoxycinnamyl alcohol	*D. officinale*	[26]
352	*trans*-syringin	*D. denneanum*	[120]
353	(1′*R*)-1′-(4-Hydroxy-3,5-dimethoxyphenyl)-1-propanol-4-O-*β*-*D*-glucoside	*D. officinale*	[26]
354	Michehedyoside C	*D. huoshanense*	[112]
355	Erigeside 2	*D. officinale*, *D. huoshanense*, *D. devonianum*	[22,26,49]
356	citrusin C	*D. devonianum*	[25]
357	isodensifloroside	*D. thyrsiflorum*	[104]

358	densifloroside	*D. thyrsiflorum*	[104]
359	Dihydroeugenol glycoside	*D. denneanum*, *D. devonianum*	[49,120]
360	erythrosyringoylglycerol-4-O-*β*-*D*-glucopyranoside	*D. huoshanense*	[22]
361	xeroboside	*D. thyrsiflorum*	[104]
**Coumarins (362-368)**			
362	Coumarin	*D. chrysanthum*, *D. thyrsiflorum*, *D. nobile*, *D. denneanum*, *D. moschatum*	[36,80,104,106,108]
363	Scoparone	*D. fimbriatum*, *D. williamsonii*, *D. palpebrae*	[57,65,111]
364	scopolin	*D. thyrsiflorum*	[104]
365	7-Methoxycoumarin-6-O-*β*-*D*-glucopyranoside	*D. officinale*	[26]
366	psoralen	*D. Sonia*	[113]
367	7-[(3,3-Dimethyloxiran-2-yl)methoxy]-8-[(3,3-dimethyloxiran-2-yl)methyl]-2*H*-1-benzopyran-2-one	*D. officinale*	[26]
368	Isopimpinellin	*D. nobile*	[1]
**Lignans (369-396)**			
369	Pinoresinol	*D. hercoglossum*, *D. devonianum*, *D. Sonia*, *D. crepidatum*, *D. officinale*, *D. aphyllum*, *D. nobile*	[1,26,67,101,113,117,119]
370	(-)-(8*R*,7'*E*)-4-hydroxy-3,3',5,5'-tetramethoxy-8,4'-oxyneolign-7'-ene-9,9'-diol 4,9-bis-O-*β*-D-glucopyranoside	*D. denneanum*	[121]
371	(-)(8*R*,7'*E*)-4-hydroxy-3,3',5,5',9'-pentamethoxy-8,4'-oxyneolign-7'-ene-9-ol 4,9-bis-O-*β*-D-glucopyranoside	*D. denneanum*	[121]
372	Magnolenin	*D. officinale*	[26]
373	(7*S*, 8*S*, 8'*R*)-Lariciresinol	*D. officinale*	[26]
374	(+)-Lyoniresin-3a-*O*-*β*-*D*-glueopyranoside	*D. officinale*	[26]
375	3-hydroxy-1-(4-hydroxy-3,5-dimethoxyphenyl)-2-[4-(3-hydroxy-1-(*E*)-propenyl)-2,6-dimethoxyphenoxy]propyl-7-O-*β*-*D*-glucopyranoside	*D. denneanum*	[120]
376	(-)-(7*S*,8*R*,7*E*)-4-hydroxy-3,3,5,5'-tetra	*D. denneanum*	[122]

	methoxy-8,4'-oxNeoLignan-7-ene-7,9,9'-triol 7,9-*di*-O-*β*-*D*-glucoside		
377	(7*R*, 8*S*, 7'*R*,8'*S*)-Syringaresinol-4-O-*β*-*D*-glucopyranoside	*D. officinale*, *D. huoshanense*	[22,26]
378	Syringaresinol-4,4'-O-*bis*-*β*-*D*-glucoside	*D. officinale*, *D. huoshanense*	[22,26]
379	(−)-(7*R*,7'*R*,8*R*,8'*S*)-4,4'-dihydroxy-3,3',5,5'-tetramethoxy-7,9'-epoxylignan-7',9-lactone	*D. denneanum*	[54]
380	Officinalioside	*D. officinale*	[26]
381	(8*S*,7'*R*, 8'*S*)-5,5'-Dimethoxy-lariciresinol	*D. officinale*	[26]
382	Conicaoside	*D. officinale*	[26]
383	icariol A_2-4-O-*β*-*D*-glucopyranoside	*D. officinale*	[26]
384	Secoisolariciresinol	*D. officinale*	[26]
385	Pinoresin-4-O-*β*-*D*-glucopyranoside	*D. officinale*	[26]
386	sesqui-illisimonans A	*D. Sonia*	[113]
387	(+)-syringaresinol-O-*β*-*D*-glucopyranoside	*D. denneanum*, *D. devonianum*	[49,120]
388	(+)-syringaresinol	*D. hercoglossum*, *D. denneanum*, *D. trigonopus*, *D. loddigesii*, *D. aphyllum*, *D. huoshanense*, *D. crystallinum*, *D. Sonia*, *D. hainanense*	[47,52,54,67,85,93,103,113,123]
389	picraquassioside C	*D. denneanum*	[120]
390	*threo*-7-O-ethyl-9-O-(4-hydroxyphenyl) propionyl-guaiacylglycerol	*D. wardianum*	[29]
391	Chaenomin B	*D. crepidatum*	[117]
392	rel-(3*R*,3'*S*,4*R*,4'*S*)-3,3',4,4'-tetrahydro-6,6'-dimethoxy[3,3'-*bi*-2*H*-benzopyran]-4,4'-diol	*D. williamsonii*	[65]
393	zhebeiresinol	*D. Sonia*	[113]
394	matairesinol	*D. sinense*	[84]
395	2,6-Dimethoxyphenyl-*β*-*D*-glucopyranoside	*D. officinale*	[26]
396	4-(*β*-*D*-Glucopyranosyl)benzyl alcohol	*D. officinale*	[26]
**Quinonoids (397-414)**			
397	2,3-dihydroxy-7-methoxy-5,8-phenanthrenedione	*D. nobile*	[1]
398	2-Hydroxy-1,7-dimethoxy-5,8-diphenan	*D. nobile*	[1]

	threnequinone		
399	6,7-dihydroxy-2-methoxy-1,4-phenanthrenedione	*D. nobile*	[35]
400	7-hydroxy-2-methoxy-1,4-phenanthrene quinone	*D. hancockii*	[59]
401	denbinobin	*D. officinale*, *D. nobile*	[1,26]
402	3-ethoxy-5-hydroxy-7-methoxy-1,4-phenanthrenequinone	*D. wardianum*	[62]
403	2-hydroxy-9,10-dihydro-5,8-phenanthrenedione	*D. nobile*	[1]
404	9,10-Dihydro-7-hydroxy-5-methoxy-1,4-phenanthrenedion	*D. officinale*	[26]
405	Denbinobin B	*D. officinale*	[26]
406	emodin	*D. chrysanthum*, *D. fimbriatum*, *D. aphyllum*	[33,71,76]
407	Rhein	*D. fimbriatum*	[111]
408	Aloe-emodin	*D. fimbriatum*	[71]
409	chrysophanein	*D. thyrsiflorum*	[104]
410	Chrysophanol-8-O-*β-D*-glucopyranoside	*D. aphyllum*	[33]
411	aloe-emodin-8-O-*β-D*-glucopyranoside	*D. aphyllum*	[33]
412	Chrysophanol-1-O-*β-D*-glucopyranoside	*D. aphyllum*	[33]
413	2,6-Dimethoxy-*p*-benzoquinone	*D. officinale*, *D. huoshanense*, *D. denneanum*	[26,52,122]
414	denbinobin	*D. wardianum*	[16]
**Phenanthrene compound (415-479)**			
415	2,4,7-Trihydroxy-9,10-dihydrophenanthrene	*D. officinale*, *D. loddigesii*, *D. huoshanense*, *D. aphyllum*, *D. devonianum*, *D. chrysotoxum*	[26,49,52,63,103,124]
416	4,7-dihydroxy-2 methoxy-9,10-dihydroxyphenanthrene	*D. nobile*, *D. aphyllum*, *D. palpebrae*, *D. gibsonii*	[1,57,68,101]
417	coelonin	*D. heterocarpum*	[40]
418	Orchinol	*D. officinale*	[26]
419	Erianthridin	*D. officinale*, *D. nobile*, *D. huoshanense*, *D. chrysotoxum*	[1,26,52,116]
420	4,7-dihydroxy-2,3-dimethoxy-9,10-dihydrophenanthrene	*D. officinale*, *D. nobile*, *D. sinense*, *D. gibsonii*	[1,26,68,70]

421	3,7-Dihydroxy-2,4-dimethoxy-9,10-dihydrophenanthrene	*D. nobile*	[1]
424	2,4,5-trihydroxy-9,10-dihydrophenanthrene	*D. chrysanthum*	[125]
425	4,5-dihydroxy-2-methoxy-9,10-dihydrophenanthrene	*D. nobile*, *D. devonianum*	[1,119]
426	2,5-dihydroxy-4-methoxy-9,10-dihydrophenanthrene	*D. chrysanthum*, *D. thyrsiflorum*, *D. nobile*, *D. aphyllum*, *D. devonianum*, *D. moschatum*, *D. strongylanthum*, *D. chrysotoxum*, *D. trigonopus*	[1,36,48,49,63,77,85,116,125]
427	4,5-dihydroxy-2,3-dimethoxy-9,10-dihydrophenanthrene	*D. pachyglossum*	[56]
428	2,5-dihydroxy-3,4-dimethoxy-9,10-dihydrophenanthrene	*D. sinense*	[70]
429	ephemeranthol C	*D. nobile*	[1]
430	2-methoxy-9,10-dihydrophenanthrene-4,5,7-triol	*D. nobile*	[1]
431	9,10-dihydro-2,7-dimethoxyphenanthrene-4,5-diol	*D. nobile*	[1]
432	2,4-dihydroxy-5-methoxy-9,10-dihydrophenanthrene	*D. chrysanthum*	[125]
433	2,5,7-trihydroxy-4-methoxy-9,10-dihydrophenanthrene	*D. formosum*, *D. sinense*, *D. bellatulum*	[42,70,96]
434	2,4-dihydroxy-7-methoxy-9,10-dihydroxyphenanthrene	*D. aphyllum*, *D. chrysotoxum*	[101,116]
435	4,7-Dihydroxy-2,3,6-trimethoxy-9,10-dihydrophenanthrene	*D. huoshanense*	[52]
436	2,7-dihydroxy-3,4,6-trimethoxy-9,10-dihydrophenanthrene	*D. sinense*	[70]
437	1,3,4-trimethoxy-9,10-dihydrophenanthrene-2,7-diol	*D. huoshanense*	[52]
438	5-methoxy-4,7,9*S*-trihydroxy-9,10-dihydrophenanthrene	*D. aphyllum*	[101]
439	5-methoxy-2,4,7,9*S*-tetrahydroxy-9,10-dihydrophenanthrene	*D. denneanum*	[121]
440	4-methoxy-2,5,7,9*S*-tetrahydroxy-9,10-dihydrophenanthrene	*D. aphyllum*	[101]
441	flavanthrinin	*D. gratiosissimum*	[37]
442	Dendrocandin P1	*D. officinale*	[26]
443	Dendrocandin P2	*D. officinale*	[26]
444	moscatin	*D. hancockii*, *D. officinale*, *D. chrysanthum*, *D. thyrsiflorum*,	[1,26,53,59,63,77,97,10

		D. loddigesii, *D. nobile*, *D. aphyllum*, *D. trigonopus*, *D. fimbriatum*, *D. denneanum*, *D. chrysotoxum*	3,106,108,116]
445	3,5-Dihydroxy-2,4-dimethoxyphenanthrene	*D. officinale*	[26]
446	2,5-Dihydroxy-3,4-dimethoxyphenanthrene	*D. officinale*	[26]
447	fimbriol B	*D. nobile*	[1]
448	lusianthrin	*D. nobile*	[1]
449	flavanthrinin	*D. nobile*	[1]
450	2,7-Dihydroxy-3,4-dimethoxyphenanthrene	*D. officinale*, *D. strongylanthum*, *D. hainanense*	[26,48,123]
451	2,4-dimethoxyphenanthrene-3,7-diol	*D. nobile*	[1]
452	7-hydroxy-2,3,4-trimethoxyphenanthrene	*D. chrysotoxum*	[45]
453	denthyrsinol B	*D. nobile*	[1]
454	denthyrsinol A	*D. nobile*	[1]
455	phoimbrtol A	*D. strongylanthum*	[48]
456	loddigesiinol A	*D. chrysanthum*, *D. stuposum*	[72,125]
457	2,7-Dihydroxy-3,4,6-trimethoxyphenanthrene	*D. chrysotoxum*	[124]
458	Chrysotoxene	*D. officinale*, *D. huoshanense*, *D. chrysotoxum*	[26,52,78]
459	2,3,4,7-Tetramethoxyphenanthrene	*D. officinale*, *D. huoshanense*	[26,52]
460	2,6-Dihydroxy-1,5,7-trimethoxyphenanthrene	*D. nobile*, *D. palpebrae*	[1,57]
461	Confusarin	*D. officinale*, *D. fimbriatum*, *D. nobile*, *D. denneanum*, *D. chrysotoxum*	[1,26,69,78,111]
462	4,5-dihydroxy-2,10-dimethoxyphenanthrene	*D. nobile*	[1]
463	1,2,6,7-tetrahydroxy-4-methoxyphenanthrene	*D. chrysotoxum*	[45]
464	2,5-Dihydroxy-4,9-dimethoxyphenanthrene	*D. hancockii*, *D. stuposum*, *D. chrysotoxum*, *D. palpebrae*	[57,59,72,116]
465	fimbriatone	*D. fimbriatum*, *D. nobile*, *D. chrysotoxum*	[1,111,116]
466	denthyrsinol C	*D. nobile*	[1]
467	denthysinol	*D. nobile*	[1]

468	dendropalpebrone	*D. palpebrae*	[57]
469	chrysotoxol A	*D. loddigesii*	[46]
470	dendronbibisline A	*D. nobile*	[1]
471	dendronbibisline B	*D. nobile*	[1]
472	5-methoxy-4,7,10*R*-trihydroxy-9,10-dihydrophenanthrene-7-O-*β*-*D*-glucopyranoside	*D. nobile*	[1]
473	9,10-Dihydro-4-methoxy-2,5-phenanthrenediol	*D. officinale*	[26]
474	loddigesinol J	*D. loddigesii*	[46]
475	4,4′,7,7′-tetrahydroxy-2,2′-dimethoxy-9,9′,10,l0′-tetrahydro-1,1′-biphenanthrene	*D. nobile*	[1]
476	phochinenin D	*D. nobile*	[1]
477	phochinenin G	*D. nobile*	[1]
478	loddigesinol I	*D. loddigesii*	[46]
479	loddigesiinol B	*D. loddigesii*	[46]
**Terpenoids (480-532)**			
480	Dendronobilin D	*D. nobile*	[126]
481	Dendronobilin E	*D. nobile*	[126]
482	Dendronobilin B	*D. nobile*	[126]
483	Dendroside F	*D. nobile*	[126]
484	Dendroside G	*D. nobile*	[126]
485	Dendronobilin L	*D. nobile*	[126]
486	Dendrodensiflorol	*D. nobile*	[126]
487	7,12-dihydroxy-5-hydroxymethyl-11-isopropyl-6-methyl-9-oxatricyclo[6.2.1.02,6]undecan-10-one-15-O-*β*-*D*-glucopyranoside C	*D. nobile*	[126]
488	10,12-dihydroxypicrotoxane	*D. nobile*	[126]
489	6*α*,10,12-trihydroxypicrotoxane	*D. nobile*	[126]
490	Dendronobiloside B	*D. nobile*	[126]
491	Dendronobiloside A	*D. nobile*	[126]
492	Dendroterpene C	*D. nobile*	[1]
493	Dendroterpene D	*D. nobile*	[1]
494	Dendronobilin H	*D. nobile*	[126]

495	Dendrobiumane A	*D. nobile*	[126]
496	10*β*,12,14-trihydroxyalloaromadendrane	*D. nobile*	[126]
497	Dendroside B	*D. nobile*	[126]
498	10*β*,13,14-trihydroxyalloaromadendrane	*D. nobile*	[126]
499	Dendroside C	*D. nobile*	[126]
500	Dendroside A	*D. nobile*	[126]
501	Dendroside D	*D. nobile*	[126]
502	Dendronobiloside C	*D. nobile*	[126]
503	Dendronobiloside D	*D. nobile*	[126]
504	cadalene-12-O-*β*-glucopyranoside	*D. nobile*	[126]
505	Dendronobilin M	*D. nobile*	[126]
506	Dendronobilin F	*D. nobile*	[126]
507	Dendronobilin J	*D. nobile*	[126]
508	Dendronobilin C	*D. nobile*	[126]
509	*α*-dihydropicrotoxinin	*D. moniliforme*	[127]
510	Nobilomethylene	*D. nobile*	[126]
511	Findlayanin	*D. nobile*	[126]
512	Dendronobilin A	*D. nobile*	[126]
513	Dendronobilin K	*D. nobile*	[126]
514	(+)*δ*-Cadinen-12,14-diol	*D. nobile*	[126]
515	Dendroside E	*D. nobile*	[126]
516	Dendronobilin G	*D. nobile*	[126]
517	Bullatantriol	*D. nobile*	[126]
518	Digiprolactone	*D. officinale*	[26]
519	(+)-(1*R*,2*S*,3*R*,4*S*,5*R*,6*S*,9*R*)-3,11,12-trihydroxypicrotoxane-2(15)-lactone	*D. nobile*	[1]
520	pimaradiene	*D. nobile*	[109]
521	(−)-(1*S*,2*R*,3*S*,4*R*,5*S*,6*R*,9*S*,12*R*)-3,11,13-trihydroxypicrotoxane-2(15)-lactone	*D. nobile*	[1]
522	Dictamnoside A	*D. officinale*	[26]
523	(+)-(1*R*,5*R*,6*S*,8*R*,9*R*)-8,12-dihydroxy-copacamphan-3-en-2-one	*D. nobile*	[1]
524	Catechol	*D. officinale*	[26]

525	corchoionoside C	*D. polyanthum*	[17]
526	fridedlin	*D. thyrsiflorum*, *D. crepidatum*	[104,110]
527	Hydroxy-(+)-epicubenol	*D. nobile*	[12]
528	Betulin	*D. williamsonii*	[65]
529	Aduncin	*D. aduncum*, *D. officinale*, *D. wardianum*	[26,107,128]
530	amotin	*D. wardianum*	[128]
531	*eqi*-*α*-amyrin	*D. nobile*	[12]
532	ursolic acid	*D. fimbriatum*	[97]
**Steroids (533-553)**			
533	(3*R*,9*R*)-9-O-*β*-*D*-Glucopyranosyl-3-hydroxy-7,8-didehydr-O-*β*-ionol	*D. officinale*	[26]
534	(3*S*,5*R*,6*R*,7*E*,9*S*)-3,5,6,9-Tetrahydroxy-7-megastigmene	*D. officinale*	[26]
535	(6*R*,9*S*)-9-Hydroxy megastigma-4,7-dien-3- one-9-O-*β*-*D*-glucopyranoside	*D. officinale*	[26]
536	diosgenin	*D. fimbriatum*	[97]
537	isonuatigentin	*D. chrysanthum*	[108]
538	β-Sitosterol	*D. officinale*, *D. moniliforme*, *D. chrysanthum*, *D. formosum*, *D. crystallinum*, *D. denneanum*, *D. thyrsiflorum*, *D. fimbriatum*, *D. nobile*, *D. chrysotoxum*, *D. williamsonii*, *D. aduncum*, *D. wardianum*, *D. aphyllum*, *D. trigonopus*, *D. devonianum*, *D. gratiosissimum*	[1,26,41,49,53,65,78,79,89,96,100,101,107,108,127-129]
539	(24*R*)-ethylcholest-5-en-3-ol-7-one	*D. crepidatum*	[117]
540	stigmasterol	*D. chrysanthum*, *D. thyrsiflorum*, *D. crystallinum*, *D. nobile*, *D. chrysotoxum*	[1,64,129-131]
541	(3*β*,5*α*,20*R*,24*R*)-ster-7-en-3-ol	*D. nobile*	[12]
542	ergosterol	*D. chrysotoxum*	[131]
543	26-O-*β*-D-glucopyranosylnuatigenin	*D. chrysanthum*	[114]
544	7-keto-*β*-sitosterol	*D. fimbriatum*, *D. officinale*	[26,97]
545	Dendrosterone	*D. ochreatum*	[132]
546	*β*-sitostenone	*D. formosum*	[96]

547	daucosterol	*D. officinale*, *D. chrysanthum*, *D. crystallinum*, *D. aduncum*, *D. aphyllum*, *D. denneanum*, *D. trigonopus*, *D. devonianum*, *D. moniliforme*, *D. thyrsiflorum*, *D. fimbriatum*, *D. williamsonii*, *D. nobile*, *D. chrysotoxum*, *D. wardianum*, *D. gratiosissimum*	[1,26,41,49,53,65,71,89,101,106-108,116,127-129]
548	stigmast-4-en-3*α*,6*β*-diol	*D. williamsonii*	[65]
549	(24*R*)-24-ethyl-5*α*-cholestan-5-ol-3,6-dione	*D. chrysotoxum*	[131]
550	ergosta-7,22-diene-3*β*,5*α*,6*β*-triol	*D. crystallinum*	[47]
551	ergosta-8(9),22-diene-3,5,6,7-tetraol	*D. williamsonii*	[65]
552	3*β*-hydroxy-5*α*,8*α*-epidioxyergosta-6,9,22-triene	*D. williamsonii*	[65]
553	5*α*,8*α*-epidioxy-24(*R*)-methycholesta-6,22-dien-3*β*-ol	*D. chrysotoxum*	[116]
554	6,9-Epoxy-ergosta-7,22-dien-3-ol	*D. officinale*	[26]
555	Cyclomargenol	*D. officinale*	[26]
556	3β,25-Dihydroxy-23-ene cyclopinacrol	*D. officinale*	[26]
**Nucleosides (557-563)**			
557	Inosine	*D. officinale*	[32]
558	Guanosine	*D. polyanthum*, *D. denneanum*, *D. officinale*	[17,115,133]
559	Adenosine	*D. nobile*, *D. polyanthum*, *D. gratiosissimum*, *D. devonianum*, *D. officinale*	[17,24,31,133,134]
560	Uridine	*D. crystallinum*, *D. officinale*	[47,133]
561	thymidine	*D. denneanum*	[120]
562	Xanthine	*D. officinale*	[26]
563	Thymidine deoxyriboside	*D. devonianum*, *D. aphyllum*, *D. officinale*	[31,33,92]
**Fluorenone compounds (564-572)**			
564	Nobilone	*D. hancockii*, *D. nobile*, *D. hainanense*, *D. chrysotoxum*, *D. palpebrae*, *D. gibsonii*	[1,45,57,59,68,123]
565	Dengibsin	*D. chrysanthum*, *D. thyrsiflorum*, *D. denneanum*, *D. chrysot*	[1,73,76,77,107,122]

		oxum, *D. nobile*, *D. aduncum*	
566	denchrysan A	*D. chrysanthum*, *D. nobile*, *D. hainanense*, *D. gibsonii*	[1,68,108,123]
567	2,4,7-Trihydroxy-5-methylflavone	*D. nobile*	[1]
568	dendroflorin	*D. chrysanthum*, *D. thyrsiflorum*, *D. nobile*, *D. denneanum*, *D. wardianum*, *D. trigonopus*, *D. chrysotoxum*, *D. palpebrae*	[1,29,53,57,76,77,116,122]
569	Dengibsinin	*D. chrysanthum*, *D. nobile*, *D. aduncum*, *D. gibsonii*	[1,68,107,114]
570	Chrysotoxone	*D. chrysanthum*, *D. aduncum*	[107,114]
571	denchrysan B	*D. thyrsiflorum*, *D. gibsonii*	[68,77]
572	Dihydrodengibsinin	*D. gibsonii*	[68]
**Others (573-649)**			
573	Icariside D_2	*D. aphyllum*	[93]
574	vanilloloside	*D. denneanum*	[115]
575	Gastrodin	*D. moniliforme*	[58]
576	3,4-dimethoxy-benzoic acid	*D. chrysotoxum*	[116]
577	syringylethanone	*D. nobile*	[1]
578	2,4,6-trimethoxyphenol-1-O-*β*-D-glucopyranoside	*D. denneanum*	[120]
579	3,4,5-Trimethoxybenzene-1-O-*β*-*D*-glucopyranoside	*D. officinale*	[26]
580	juniperoside	*D. nobile*	[1]
581	4-Allyl-2,6-dimethoxyphenyl glucoside	*D. officinale*	[26]
582	4-Hydroxymethyl-2,6-dimethoxyphenyl-*β*-*D*-glucopyranoside	*D. officinale*	[26]
583	Phenyl *β*-*D*-glucoside	*D. officinale*	[26]
584	Palmitic acid	*D. chrysanthum*	[114]
585	octacosanoicacid	*D. denneanum*	[100]
586	n-triaconatanoicacid	*D. denneanum*	[100]
587	n-dotriacontanoic acid	*D. fimbriatum*	[79]
588	1-Dodecanol	*D. aphyllum*	[101]
589	1-tetracosanol	*D. nobile*	[1]
590	1-hexacosanol	*D. nobile*	[1]

591	2-phenylethyl-*β*-*D*-glucopyranoside	*D. aphyllum*	[33]
592	Dendrodevonic acid A	*D. devonianum*	[119]
593	Dendrodevonic acid B	*D. devonianum*	[119]
594	hexadecanoic acid 2,3-dihydroxypropy l ester	*D. hercoglossum*	[28]
595	Heptadecanoic acid 2,3-dihydroxy-pro pyl ester	*D. chrysotoxum*	[135]
596	*α*-tocopherolquinone	*D. nobile*	[12]
597	2-(3,15-dihydroxy-3,7,11,15-tetramethyl hexadecyl)-3,5,6-trimethyl-2,5-cyclohex adiene-1,4-dione	*D. nobile*	[12]
598	Blumenol A	*D. hainanense*	[123]
599	(+)-dehydrovomifoliol	*D. hainanense*, *D. nobile*	[14,123]
600	5-Hydroxymethylfurfural	*D. huoshanense*, *D. denneanu m*	[52,98]
601	5-acetoxymethylfuraldehyde	*D. denneanum*	[98]
602	3-(4'-formylphenoxy)-4-methoxybenzal dehy-de	*D. nobile*	[12]
603	ayapin	*D. thyrsiflorum*, *D. fimbriatum*	[104,111]
604	dibutyl phthalate	*D. thyrsiflorum*, *D. nobile*	[1,129]
605	Methyl 2-nonanoate-3-undecanoate-cyc lohexyl-1,4-diene ester	*D. nobile*	[12]
606	bis(2-ethylhexyl) phthalate	*D. crepidatum*, *D. denneanum*	[98,117]
607	moellendorffiline	*D. nobile*	[1]
608	(6*aR*,11*aR*)-medicarpin	*D. stuposum*	[72]
609	dehydrodiconiferyl alcohol-4-*β*-*D*-gluc oside	*D. nobile*	[1]
610	Spiropreussomerin A	*D. formosum*	[96]
611	Daldinium A	*D. chrysotoxum*	[118]
612	(−)-6*R*-signatone	*D. signatum*	[10]
613	(9*Z*,12*Z*)-methyl octadeca-9,12-dienoat e	*D. sinense*	[84]
614	13-hydroxy-9(*Z*),11(*E*)-octadecadienoic acid (1)-coriolic acid	*D. moschatum*	[36]
615	(-)-shikimic acid	*D. fimbriatum*, *D. gratiosissim um*	[24,71]
616	methyl pyroglutamate	*D. huoshanense*	[22]

617	*D*-allitol	*D. aphyllum*	[93]
618	1-Palmitoylglycerol	*D. thyrsiflorum*	[104]
619	dl-*α*-tocopherol	*D. nobile*	[12]
620	3,4-dimethoxy-benzoic acid methyl ester	*D. chrysotoxum*	[116]
621	Auriculatum A	*D. officinale*	[26]
622	Urticifolene	*D. officinale*	[26]
623	n-hexacos-5,8,11-trienoicacid	*D. denneanum*	[100]
624	Spermidine	*D. officinale*	[26]
625	lasiodiplodin	*D. sinense*	[70]
626	Decumbic acid A	*D. nobile*	[1]
627	Decumbic acid B	*D. nobile*	[1]
628	Peroxiatractylenolide III	*D. officinale*	[26]
629	3*H*-cyclopenta[*de*]-2-benzopyran-3-one	*D. devonianum*	[119]
630	(6*S*,9*R*)-blumenol C	*D. formosum*	[96]
631	3,9-dihydroxy-megastigma-5-ene	*D. sinense*	[84]
632	2,2'-oxybis (1,4)-di-tertbutylbenze	*D. nobile*	[12]
633	(4*S*,5*S*,6*Z*,8*E*)-5-hydroxydeca-6,8-dien-4-olide	*D. formosum*	[96]
634	(−)-decumbic acid	*D. nobile*	[1]
635	2-Methoxyphenyl-1-O-*β*-*D*-apiosyl-(1→2)-*β*-*D*-glucoside	*D. officinale*	[26]
636	n-butyl-*α*-*D*-fructofuranoside	*D. gratiosissimum*	[24]
637	shikimic acid n-butyl ester	*D. gratiosissimum*	[24]
638	Dendrocandin R	*D. officinale*	[26]
639	2,3,4,9-tetrahydro-1*H*-pyrido[3,4-b]indole-3-carboxylic acid	*D. devonianum*	[31]
640	3,5-Dimethoxy-Phenethylamines	*D. officinale*	[136]
641	(±)-nobilol A	*D. nobile*	[137]
642	Dendroboside	*D. officinale*	[26]
643	3,4,5-Trimethoxyphenyl-1-O-*β*-*D*-apiosyl-(1→2)-*β*-*D*-glucopyranoside	*D. officinale*	[26]
644	9-*β*-*D*-allofuranulsylguanine	*D. denneanum*	[115]
645	Dendrodevonin A	*D. devonianum*	[119]

646	Dendrodevonin B	*D. devonianum*	[119]
647	denobilone C	*D. nobile*	[1]
648	denobilone B	*D. nobile*	[1]
649	dendroside	*D. nobile*	[1]

Uncategorized References

1. Ling-Hu Chu Gr-h, Q.L.-K. Research progress on chemical constituents and pharmacological effects of *Dendrobium nobile*. *Chinese Traditional and Herbal Drugs* **2021**, *52*, 7693-7708, doi:10.7501/j.issn.0253-2670.2021.24.032.
2. Inubushi Y , N.J. Structure of dendrine. *Tetrahedron letters* **1965**, *5*, 2723-2728.
3. Qun Fang LIU, W.M.Z. A New Dendrobine-Type Alkaloid from *Dendrobium nobile*. *Chinese Chemical Letters* **2003**, *14*, 278-279.
4. Inubushi, Y., Ishii, H. , Yasui, B. , Konita, T. , Harayama, T. Isolation and Characterization of Alkaloids of the Chinese Drug \"Chin-Shih-Hu.\". *CHEMICAL & PHARMACEUTICAL BULLETIN* **1964**, *12*, 1175-1180.
5. Hedman K, L.K. Studies on Orchidaceae Alkaloids. XXVII. Quaternary Salts of the Dendrobine Type from *Dendrobium nobile* Lindl. *Acta Chem Scand* **1972**, *26*, 3177-3180, doi:https://doi.org/10.3891/acta.chem.scand.26-3177.
6. Yang, D.; Cheng, Z.-Q.; Yang, L.; Hou, B.; Yang, J.; Li, X.-N.; Zi, C.-T.; Dong, F.-W.; Liu, Z.-H.; Zhou, J., et al. Seco-Dendrobine-Type Alkaloids and Bioactive Phenolics from *Dendrobium findlayanum*. *Journal of Natural Products* **2018**, *81*, 227-235, doi:10.1021/acs.jnatprod.7b00150.
7. WANG H, Z.T., CHE C T. Dendrobine and 3-hydroxy-2-oxodendrobine from *Dendrobium nobile*. *Journal of Natural Products* **1985**, *48*, 796-801.
8. Okamoto T, N.M., Onaka T. Further Studies on the Alkaloidal Constituents of *Dendrobium nobile* (Orchidaceae)-Structure Determination of 4-Hydroxy-dendroxine and Nobilomethylene. *Chemical & Pharmaceutical Bulletin* **1972**, *20*, 418-421, doi:https://doi.org/10.1248/cpb.20.418.
9. Okamoto T, N.M., Onaka T. The structure of dendramine (6-oxydendrobine) and 6-oxydendroxine the fourth and fifth alkaloid from *Dendrobium nobile*. *Chemical & Pharmaceutical Bulletin* **1966**, *14*, 676-680, doi:https://doi.org/10.1248/cpb.14.676.
10. Khumploy, P.; Raksat, A.; Choodej, S.; Aree, T.; Ngamrojanavanich, N.; Pudhom, K. Picrotoxane sesquiterpene and α-pyrone derivative from *Dendrobium signatum* and their free radical scavenging potency. *Journal of Natural Medicines* **2021**, *75*, 967-974, doi:10.1007/s11418-021-01547-5.
11. Okamoto T, N.M., Onaka T. The Structure of Dendroxine The Third Alkaloid from *Dendrobium nobile*. *Chemical & Pharmaceutical Bulletin* **1966**, *14*, 672-675, doi:https://doi.org/10.1248/cpb.14.672.
12. Lei, H.; Zou, S.; Lin, J.; Zhai, L.; Zhang, Y.; Fu, X.; Chen, S.; Niu, H.; Liu, F.; Wu, C., et al. Antioxidant and anti-inflammatory activity of constituents isolated from *Dendrobium nobile* (Lindl.). *Frontiers in Chemistry* **2022**, *10*, doi:10.3389/fchem.2022.988459.

13. Gui-Yuan Liu, L.T., Lei Cheng, Li-Sheng Ding, Yan Zhou, Yun Deng, Yu-Qi He,Da-Le Guo, Shi-Ji Xiao. Dendrobine-type alkaloids and bibenzyl derivatives from *Dendrobium findlayanum*. *Fitoterapia* **2020**, *142*, doi:10.1016/j.fitote.2020.104497.
14. Wang, P.; Chen, X.; Wang, H.; Huang, S.; Cai, C.; Yuan, J.; Zhu, G.; Xu, X.; Mei, W.; Dai, H. Four New Picrotoxane-Type Sesquiterpenes From *Dendrobium nobile* Lindl. *Frontiers in Chemistry* **2019**, *7*, doi:10.3389/fchem.2019.00812.
15. Meng, C.-W.; He, Y.-L.; Peng, C.; Ding, X.-J.; Guo, L.; Xiong, L. Picrotoxane sesquiterpenoids from the stems of *Dendrobium nobile* and their absolute configurations and angiogenesis effect. *Fitoterapia* **2017**, *121*, 206-211, doi:10.1016/j.fitote.2017.07.017.
16. Zhang, C.; Liu, S.-J.; Yang, L.; Yuan, M.-Y.; Li, J.-Y.; Hou, B.; Li, H.-M.; Yang, X.-Z.; Ding, C.-C.; Hu, J.-M. Sesquiterpene amino ether and cytotoxic phenols from *Dendrobium wardianum* Warner. *Fitoterapia* **2017**, *122*, 76-79, doi:10.1016/j.fitote.2017.08.015.
17. Mei Yuan, Y.Q.-h., Yang Pei-ming, Kong De-yun, Cheng Liang. Chemical Constituents of *Dendrobium polyanthum* Lindl. *Chinese Journal of Pharmaceuticals* **2014**, *45*, 224-228, doi:https://doi.org/10.16522/j.cnki.cjph.2014.03.014.
18. Hinton, B.L.K.L.N.J.G.C.G.M. Studies on Orchidaceae Alkaloids. III. The Alkaloids in *Dendrobium primulinum* Lindl. and *Dendrobium chrysanthum* Wall. *Acta Chemica Scandinavica* **1965**, *19*, 1607-1611.
19. Xu, X.; Chen, X.; Yang, R.; Li, Z.; Zhou, H.; Bai, Y.; Yu, M.; Li, B.; Ding, G. Crepidtumines A and B, Two Novel Indolizidine Alkaloids from *Dendrobium crepidatum*. *Scientific Reports* **2020**, *10*, doi:10.1038/s41598-020-66552-2.
20. Ding, X.-Q.; Zou, Y.-Q.; Liu, J.; Wang, X.-C.; Hu, Y.; Liu, X.; Zhang, C.-F. Dendrocrepidamine, a novel octahydroindolizine alkaloid from the roots of *Dendrobium crepidatum*. *Journal of Asian Natural Products Research* **2021**, *23*, 1085-1092, doi:10.1080/10286020.2021.1935891.
21. Hu Yang, Z.C.-f., Zhao Xin, Wang Yue, Feng De-qiang, Zhang Mian, Xie Hai-feng. (±)-Homocrepidine A, a Pair of Anti-inflammatory Enantiomeric Octahydroindolizine Alkaloid Dimers from *Dendrobium crepidatum*. *Journal of Natural Products* **2015**, *79*, 252-256, doi:10.1021/acs.jnatprod.5b00801.
22. Zhao Hong-Su, X.F.-Q., Chen Xiao-Xiao, Hu Jiang-Miao, Zeng Fan-Jun, Peng Dai-Yin, Wu De-Ling. Chemical constituents of *Dendrobium huoshanense* C. Z.Tang et S. J. Cheng. *Natural Product Research And Development* **2021**, *33*, 1491-1498.
23. Liu WH, H.Y., Zhan ZJ. Moniline, a new alkaloid from *Dendrobium moniliforme*. *Journal of Chemical Research* **2007**, *6*, 317-318.
24. Jiangmiao, G.W.Y.L.L.H.L.C.L.S.H. Phytochemical Study of *Dendrobium gratiosissimum*. *Modern Chinese Medicine* **2015**, *17*, 311-314.
25. Shun-Xing, M.Z.-X.D.H.-L.W.C.-L.G. Chemical Constituents of *Dendrobium devonianum*. *Chinese Pharmaceutical Journal* **2013**, *48*, 855-859.
26. Zhang Shi-Yu, G.X.-J., Zhou Xin, Mu Ying-Su, Chen Hua-Guo, Liang Kang, Zhao Chao. Research progress on chemical components and pharmacological effects of *Dendrobium officinale*. *Journal of Gansu Agricultural University* **2023**.
27. Guan huijuan, Z.x., Tu fengjuan, Yao xinsheng. Study on chemical constituents of *Dendrobium officinale*. *Chinese Traditional and Herbal Drugs* **2009**, *40*, 1873-1876.

28. Cheng Lei CZ-Y, S.Z.-M., Zhang Mao-Sheng, Li Xiao-Fei, Zhang Jian-Yong, Xiao Shi-Ji. Chemical constituents of *Dendrobium hercoglossum*. *Chinese Traditional and Herbal Drugs* **2020**, *51*, 3126-3130, doi:https://doi.org/10.7501/j.issn.0253-2670.2020.12.002.
29. LI Hanfen, Q., ZHOU Zeqin, YANG Liu, HU Jiangmiao. Phenolic chemical components from *Dendrobium wardianum*. *Chinese Traditional and Herbal Drugs* **2021**, *55*, 711-719, doi:https://doi.org/10.7501/j.issn.0253-2670.2024.03.003.
30. Wang Dai-Fang CG-X, Z.N.-Y., Zhang Ting, Xu Hong. Study on chemical constituents in stems of *Dendrobium nobile*. *Chinese Traditional and Herbal Drugs* **2012**, *43*, 1492-1495.
31. Jin-Ping, Z.A.-L.Y.M.X.H.-H.S. Constituents of *Dendrobium devonianum* and their antioxidant activity. *China Journal of Chinese Materia Medica* **2013**, *38*, 844-847.
32. Zhang chunhua, X.q., Zeng lei, Wang song, Luo qingwen, Chen yingle. Chemical Constituents from Leaves of *Dendrobium officinale*. *Forestry and envitonmental science* **2020**, *36*, 30-34.
33. He Ru-shang, W.M., Li Shi-wei, Xue Bian-xia, Zhang Li-hua, Wu Hong-hua. Chemical constituents from *Dendrobium aphyllum*. *Chinese Traditional Patent Medicine* **2023**, *45*, 3633-3638.
34. Meng weitong, M.x., Niu liting, Zhang sisi, Ouyang chunjie, Ding chunhua, Zhu lingjuan, Zhang xue. A new bibenzyl dervative from stems of *Dendrobium officinale*. *china Journal of Chinese Materia Medica* **2023**, *48*, 700-706.
35. Zhou Xue-Ming, Z.C.-J., Gan Li-She, Chen Guang-Ying, Zhang Xiao-Peng, Song Xiao-Ping, Li Gao-Nan, Sun Chong-Ge. Bioactive Phenanthrene and Bibenzyl Derivatives from the Stems of *Dendrobium nobile*. *Journal of Natural Products* **2016**, *79*, 1791-1797, doi:10.1021/acs.jnatprod.6b00252.
36. Yang Zong-Yu, Z.Y., Yang Jin, Luo Wan-Li, Zhang Mao-Sheng, Wang Gang, Sun Cheng-Xin, Dong Min-Jian, Xiao Shi-Ji. Chemical constituents from *Dendrobium moschatum*. *Chinese Traditional Patent Medicine* **2022**, *44*, 3517-3521.
37. Sun Jia-Wei, L.J.-M., Chen Ri-Dao, Liu Yu-Yu, Li Yan, Cen Shan, Chen Xiao-Mei, Guo Shun-Xing, Dai Jun-Gui. Study on chemical bibenzyls in *Dendrobium gratiosissimum*. *China Journal of Chinese Materia Medica* **2020**, *45*, 4929-4937.
38. Ren Gang, C.Y.-T., Ye Jin-Bao, Zhong Guo-Yue, Xiao Chuan-Yun, Deng Wen-Zan, Chen Yun-Long. Phytochemical investigation of leaves of *Dendrobium officinale*. *Chinese Traditional and Herbal Drugs* **2020**, *51*, 3637-3644, doi:10.7501/j.issn.0253-2670.2020.14.005.
39. Jian-Ping, S.S.-Q.J.H.L.Q.-M.Z.X.-Q.L. Study on chemical constituents of *Dendrobium huoshanense* stems and their anti-inflammatory activity. *China Journal of Chinese Materia Medica* **2020**, *45*, 3452-3458.
40. Yang Xiao-Bei, Y.S., Hu Jiang-Miao, Zhou Jun. Chemical constituents from *Dendrobium heterocarpum* Lindl. . *Natural Product Research And Development* **2019**, *31*, 1745-1752.
41. Yang Dan, C.Z.-Q., Ding Zhong-Tao, Zhou Jun, Hu Jiang-Miao. Chemical constituents of *Dendrobium crystallinum*. *Guihaia* **2017**, *37*, 1182-1186.
42. Shang Zhi-Mei, C.L., Liu Gui-Yuan, Zhang Mao-Sheng, Li Xiao-Fei, Xiao Shi-Ji. Chemical constituents of *Dendrobium bellatulum*. *Chinese Traditional and Herbal Drugs* **2019**, *50*, 2036-2040.
43. Yupeng, Z.C.L. Research Progresson Chemical Constituents and Pharmacological Effects of *Dendrobium polyanthum*. *Industrial Microbiology* **2024**, *54*, 48-50, doi:10.3969/j.issn.1001-6678.2024.02.016.

44. Yang Dan, L.L.-Y., Cheng Zhong-Quan, Xu Feng-Qing, Fan Wei-Wei, Zi Cheng-Ting, Dong Fa-Wu, Zhou Jun, Ding Zhong-Tao, Hu Jiang-Miao. Five new phenolic compounds from *Dendrobium aphyllum*. *Fitoterapia* **2015**, *100*, 11-18, doi:10.1016/j.fitote.2014.11.004.
45. Liu Ying, Z.J.Q., Zhan Rui, Chen Ye-Gao. Isopentenylated Bibenzyls and Phenolic Compounds from *Dendrobium chrysotoxum* Lindl. *Chemistry & Biodiversity* **2022**, *19*, doi:10.1002/cbdv.202200259.
46. Jun, L.X.-W.C.H.-P.H.W.-B.Y.W.-L.N.F.-Y.H.Z.-W.H.H.-Y.W. Polyphenols from *Dendrobium loddigesii* and their biological activities. *Acta Scientiarum Naturalium Universitatis Sunyatseni* **2019**, *58*, 96-102.
47. Wang Lei, Z.C.-F., Wang Zheng-Tao, Zhang Mian, Xu Luo-Shan. Chemical constituents of *Dendrobium crystallinum*. *Chinese Traditional and Herbal Drugs* **2011**, *42*, 31-33.
48. Luo Wan-li, W.M.y., Yang Zong-yu, Zhang Mao-sheng, Wang Gang, Sun Cheng-xin, Dong Min-jian, Xiao Shi-ji. Chemical constituents from *Dendrobidium strongylanthum*. *Chinese Traditional Patent Medicine* **2022**, *44*, 3192-3195.
49. Guangyu, W.h.L.M.H.J.C.L.M. Research Advances of *Dendrobium Devonianum*. *CHINESE ARCHIVES OF TRADITIONAL CHINESE MEDICINE* **2014**, *32*, 2732-2735.
50. Deng Guang-Hui, Y.N.-J., Wang Yan-Yan, Ye Meng-Juan, Cai Xiao, Zhang Xiao-Qian, Peng Dai-Yin. Research progress on effect and mechanism of *Dendrobium huoshanense* and its active components against liver diseases. *Chinese Traditional and Herbal Drugs* **2022**, *53*, 6959-6967, doi:10.7501/j.issn.0253-2670.2022.21.034.
51. Shun-Xing, C.X.-M.W.C.-L.Y.J.-S.G. Research Progress on Chemical Composition and Chemical Analysis of *Dendrobium officinale*. *Chinese Pharmaceutical Journal* **2013**, *48*, 1634-1640.
52. Cai Ming, C.J.-W., Yang Qing-Shan, Yu Jiao, Xie Hui-Qun, Han Lan, Peng Dai-Yin. Review of chemical composition and pharmacological effects of *Dendrobium huoshanense* and prediction of its Q-markers. *China Journal of Chinese Materia Medica* **2024**, 10.19540/j.cnki.cjcmm.20240704.201, 1-16, doi:10.19540/j.cnki.cjcmm.20240704.201.
53. Zhang Ting, Z.C.-F., Wang Zheng-Tao, Xu Luo-Shan. Studies on Chemical Constituents of *Dendrobium trigonopus* Rchb. f. *Chinese Journal Of Natural Medicines* **2005**, *3*, 28-30.
54. Yang Lian, Z.Q.-M., Peng Cheng, Guo Li, Zhang Ting-Mo, Xiong Liang. Chemical constituents from *Drobium aurantiacum* var. *denneanum*. *Chinese Traditional and Herbal Drugs* **2015**, *46*, 2193-2197.
55. Zhang Xue, X.J.-K., Wang Jue, Wang Nai-Li, Kurihara Hiroshi, Kitanaka Sumumu, Yao Xin-Sheng. Bioactive Bibenzyl Derivatives and Fluorenones from *Dendrobium nobile*. *Journal of Natural Products* **2007**, *70*, 24-28.
56. Warinhomhoun S, M.C., Buranasudja V, Mekboonsonglarp W, Rojsitthisak P, Likhitwitayawuid K, Sritularak B. Antioxidant Activities and Protective Effects of Dendropachol, a New Bisbibenzyl Compound from *Dendrobium pachyglossum*, on Hydrogen Peroxide-Induced Oxidative Stress in HaCaT Keratinocytes. *Antioxidants* **2021**, *10*, doi:10.3390/antiox10020252.
57. Kyokong, N.; Muangnoi, C.; Thaweesest, W.; Kongkatitham, V.; Likhitwitayawuid, K.; Rojsitthisak, P.; Sritularak, B. A new phenanthrene dimer from *Dendrobium palpebrae*. *Journal of Asian Natural Products Research* **2018**, *21*, 391-397, doi:10.1080/10286020.2018.1429416.
58. Yu muyuan Hj, H.y. Study on chemical constituents of *Dendrobium moniliforme*. *Energy and Environment* **2014**, *3*, 35-36.

59. Shang Zhi-mei XD, C.L., Liu Gui-Yuan, Zhang Mao-Sheng, Zhang Jian-Yong, Li Xiao-Fei, Xiao Shi-Ji. Chemical constituents from *Dendrobium hancockii*. *Chinese Traditional and Herbal Drugs* **2019**, *50*, 3760-3763, doi:https://doi.org/10.7501/j.issn.0253-2670.2019.16.004.
60. Wang, Y.-H. Traditional uses, chemical constituents, pharmacological activities, and toxicological effects of *Dendrobium* leaves: A review. *Journal of Ethnopharmacology* **2021**, *270*, doi:10.1016/j.jep.2021.113851.
61. Li Chun-yan, L., Chen Ye, Zheng Jia-wen, Wang Jun. Chemical Components of *Dendrobium loddigesii*. *Acta Scientiarum Naturalium Universitatis Sunyatseni* **2013**, *52*, 73-76, doi:https://doi.org/10.13471/j.cnki.acta.snus.2013.03.011.
62. Li Jing-juan RF-c, H.J.-m., Zhou Jun. Chemical constituents and cytotoxic activities of *Dendrobium wardianum*. *Chinese Traditional and Herbal Drugs* **2020**, *51*, 1819-1824, doi:https://doi.org/10.7501/j.issn.0253-2670.2020.07.016.
63. Zhang chaofeng, S., Huang weihua, Wang lei, Wang zhengtao, Xu luoshan. Phenolic components from herbs of *Dendrobium aphyllum*. *Zhongguo China journal of Chinese materia medica* **2008**, *33*, 2922-2925.
64. Wang Lei ZC-F, W.Z.-T., Zhang Mian, Shao Li, Xu Luo-Shan. Studies on chemical constituents of *Dendrobium crystallinum*. *China journal of Chinese materia medica* **2008**, *33*, 1847-1848, doi:https://doi.org/10.1007/s10600-007-0234-7.
65. Yang, M.; Zhang, Y.; Chen, L.; Chen, Y. A new (propylphenyl)bibenzyl derivative from *Dendrobium williamsonii*. *Natural Product Research* **2017**, *32*, 1699-1705, doi:10.1080/14786419.2017.1396599.
66. Shah, B.Z.M.W.Z.T.X.L. Chemical Constituents of *Dendrobium moniliforme*. *Acta Botanica Sinica* **2004**, *46*, 124-126.
67. Guo Dongsheng Yl, S.X., Mei Wenli, Chen Huiqin, Dai Haofu. Chemical Constituents from Aerial Parts of *Dendrobium hercoglossum* (Orchidaceae) and Their Biological Activities. *Journal of Tropical and Subtropical Botany* **2024**, *32*, 287-293, doi:https://doi.org/10.11926/jtsb.4736.
68. Thant, M.T.; Chatsumpun, N.; Mekboonsonglarp, W.; Sritularak, B.; Likhitwitayawuid, K. New Fluorene Derivatives from *Dendrobium gibsonii* and Their α-Glucosidase Inhibitory Activity. *Molecules* **2020**, *25*, doi:10.3390/molecules25214931.
69. Xu, G.-X.M.T.-S.W.L.Y.Y.P.G.-J.X.L.-S. Studies on Chemical Constituents of *Dendrobium chryseum*. *Journal of Chinese Pharmaceutical Sciences* **1998**, *7*, 52-54.
70. Tan Cai-Yin, M.-L., Zhao You-Xing, Huang Sheng-Zhuo, Kong Fan-Dong, Yang Ning-Ning, Song Xi-Qiang, Dai Hao-Fu. Chemical Constituents from *Dendrobium sinense*. *Journal of Tropical and Subtropical Botany* **2017**, *25*, 189-194, doi:https://doi.org/10.11926/jtsb.3668.
71. Bi zhiming, W., Zhang mian, Xu deran, Xu luoshan, Xu guojun. Studies on Chemical Constituents of *Dendrobium fimbriatum* Hook. (II). *Journal of China Pharmaceutical University* **2001**, *32*, 421-422.
72. QIN Zemin, Z., FU Huan, HU Jun, YANG Minghui. Study on Chemical Constituents of *Dendrobium Stuposum*. *Chinese Journal of Ethnomedicine and Ethnopharmacy* **2019**, *28*, 19-22.
73. Hu, J.; Fan, W.; Dong, F.; Miao, Z.; Zhou, J. Chemical Components of *Dendrobium chrysotoxum*. *Chinese Journal of Chemistry* **2012**, *30*, 1327-1330, doi:10.1002/cjoc.201100670.

74. Xiao Shi-Ji LZ, Z.M.-S., Chen Yong-Zheng, Nie Xu-Qiang, Zhang Jian-Yong, He Yu-Qi, Shi Jing-Shan. A new bibenzyl compound from *Dendrobium nobile*. *Acta Pharmaceutica Sinica* **2016**, *51*, 1117-1120, doi:https://doi.org/10.16438/j.0513-4870.2016-0059.
75. Ye qinghua, Z., Qin guowei. Study on chemical constituents of *Dendrobium chrysanthum*. *Chinese Traditional and Herbal Drugs* **2004**, *35*, 1101-1103.
76. Yang Li, W., Bi Zhi-Ming, Lin Ping, Wang Zheng-Tao, Xu Luo-Shan. Studies on Chemical Constituents of *Dendrobium chrysanthum*. *Chinese Journal of Natural Medicines* **2004**, *2*, 280-282.
77. Zhang guangnong, Z., Wang zhengtao, Xu luoshan. Studies on Chemical Constituents of *Dendrobium thyrsiflorum* Rchb.f (Ⅰ). *Chinese Journal Of Natural Medicines* **2004**, *2*, 78-82.
78. Ma guoxiang, X., Xu luoshan, Wang zhengtao, T Kickchi. Studies on Chemical Constituents of *Dendrobium chrysotoxum* Lindl. *Acta Pharmaceutica Sinica* **1994**, *29*, 763-766, doi:https://doi.org/10.16438/j.0513-4870.1994.10.008.
79. Bi Zhi Ming, Y.Y.S., Wang Zheng-Tao, Gong Yan-Qing, He Ju-Xiu, Tadato Tani. Chemical Constituents of *Dendrobium fimbriatum* Hook. (I). *Journal of China Pharmaceutical University* **2001**, *32*, 200-202.
80. Xu Li, W.-R., Guo Li, Zhang Ting-Mo. Chemical constituents from *Dendrobium nobile*. *Chinese Traditional Patent Medicine* **2018**, *40*, 1110-1112, doi:https://doi.org/10.3969/j.issn.1001-1528.2018.05.021.
81. Zhang Maosheng, L., Li Xiaofei, Zhang Jianyong, Nie Xuqiang, Guo Dale, Xiao Shiji. Bibenzyl Derivatives from *Dendrobium nobile*. *Chinese Journal of Organic Chemistry* **2019**, *39*, 3289-3293, doi:https://doi.org/10.6023/cjoc201903035.
82. Zhu, L.-J.; Wang, M.-Q.; Qin, Y.; Wang, M.-N.; Zhang, G.-Q.; Niu, L.-T.; Chen, J.-B.; Zhang, X.; Yao, X.-S. Two new dibenzyl derivatives from the stems of *Dendrobium catenatum*. *Journal of Asian Natural Products Research* **2020**, *23*, 955-960, doi:10.1080/10286020.2020.1826937.
83. Cheng, L.; Guo, D.-L.; Zhang, M.-S.; Linghu, L.; Fu, S.-B.; Deng, Y.; He, Y.-Q.; Xiao, S.-J. Dihydrophenanthrofurans and bisbibenzyl derivatives from the stems of *Dendrobium nobile*. *Fitoterapia* **2020**, *143*, doi:10.1016/j.fitote.2020.104586.
84. Cai Caihong, T., Chen Huiqin, Wang Hao, Mei Wenli, Song Xiqiang, Dai Haofu. Chemical constituents from *Dendrobium sinense* (II). *Guihaia* **2020**, *40*, 1368-1374, doi:https://doi.org/10.11931/guihaia.gxzw201907003.
85. Hu, J.-M.; Chen, J.-J.; Yu, H.; Zhao, Y.-X.; Zhou, J. Two novel bibenzyls from *Dendrobium trigonopus*. *Journal of Asian Natural Products Research* **2008**, *10*, 647-651, doi:10.1080/10286020802133605.
86. Cao Yang, D., Li Bo-Teng, Zhang Shu-Hua, Liang Chong, Zhang Guo-Gang. Isolation and identification of ethanol extract from *Dendrobium officinale* Kimura et Migo. *Chinese Journal of Medicinal Chemistry* **2019**, *29*, 385-389, doi:https://doi.org/10.14142/j.cnki.cn21-1313/r.2019.05.010.
87. Zhou Yu-juan, W.J.-h., Xu Hong, Chou Gui-xin, Wang Zheng-tao. Bibenzyls from *Dendrobium officinale*. *China Journal of Chinese Materia Medica* **2021**, *46*, 3853-3858.
88. Wang Jing-Wen, X.-N., Zhang Hai-Lang, Lei Guo-Ping, Xu Wei, Yang Yun-Zhi. Flavonoids from *Dendrobium fimbriatum* and its protective effect on corticosterone-induced PC12 cell damage. *Central South Pharmacy* **2024**, *22*, 974-979, doi:https://doi.org/10.7539/j.issn.1672-2981.2024.04.023.

89. Wang Min, Z.C.-F., Wang Zheng-Tao, Zhang Mian. Studies on constituents of *Dendrobium gratiosissimum*. *China Journal of Chinese Materia Medica* **2007**, *32*, 701-703.
90. Xiaobo, F.H.P.C.L. Study on the separation and identification of flavone *C*-glycosides from the leaves of *Dendrobium officinale* and their inhibitory activities to α-gulcosidase. *China Pharmacy* **2022**, *33*, 2187-2191, doi:10.6039/j.issn.1001-0408.2022.18.03.
91. Wei Ze-Yuan, L.J.-J., Jin Chuanshan, Xia Hong. Chemical Constituents from n-butanol Extracts of *Dendrobium officinale*. *Modern Chinese Medicine* **2013**, *15*, 1042-1045, doi:10.13313/j.issn.1673-4890.2013.12.014.
92. Zhou Jia, Z.X.-l., Liang Cheng-qin, Su Xiao-jian, Li Bo-lin, Qin Ya-yan, Wu Yuan-e. Chemical constituents of *Dendrobium officinale*. *Chinese Traditional and Herbal Drugs* **2015**, *46*, 1292-1295.
93. Shao Li, H.W.-H., Zhang Zhao-Feng, Wang Lei, Zhang Mian, Wang Zheng-Tao. Study on chemical from stem of *Dendrobium aphyllum*. *China Journal of Chinese Materia Medica* **2008**, *33*, 1693-1695.
94. Zhou Yu-Juan, W.J.-H., Xue Ya-Fu, Xu Hong, Chou Gui-Xin, Wang Zheng-Tao Study on chemical constituents from active ethyl acetate fraction of *Dendrobium officinale Chinese Traditional and Herbal Drugs* **2021**, *52*, 5218-5225, doi:10.7501/j.issn.0253-2670.2021.17.013.
95. Li Yan, W.C.-L., Wang Fang-Fei, Dong Hai-Ling, Guo Shun-Xing, Yang Jun-Shan, Xiao Pei-Gen. Phenolic Components and Flavanones from *Dendrobium candidum*. *Chinese Pharmaceutical Journal* **2010**, *45*, 975-979.
96. Chi Li-Hang, C.H.-Q., Huang Sheng-Zhuo, Wu Fei, Mei Wen-Li, Song Xi-Qiang, Dai Hao-Fu. Chemical constituents from the stems and leaves of *Dendrobium formosum* and their biological activities. *Chinese Traditional Patent Medicine* **2024**, 1-6.
97. Liu Sha-Sha, L.-R., Sun Jing-Xian, Wang Rui-Sheng, Shao Jin-Liang, Liu Hong-Cheng, Huang Xiang-Zhong, Li Yan-Hong. Chemical constituents from the ethanol extracts of *Dendrobium fimbriatum* Hook. *Journal of Yunnan Nationalities University* **2022**, *31*, 375-380, doi:https://doi.org/10.3969/j.issn.1672-8513.2022.04.001.
98. Xu Li, L.X.-W., Wang Jiang-Rui, Ye Qiang, Guo Li, Zhang Ting-Mo. Chemical constituents of *Dendrobium aurantiacum* Rchb. f. var. *denneanum* (Kerr) Z. H. Tsi. *Chinese Traditional Patent Medicine* **2015**, *37*, 1010-1012.
99. Wang Jun-Hao, L.W.-X., Wu Si-Jia, Li Jian, Wang Zheng-Tao, Xu Hong. A new *allo*-aromadendrane sesquiterpene from *Dendrobium nobile*. *China Journal of Chinese Materia Medica* **2023**, *48*, 6088-6092.
100. Minghui, B.Y.Z.X.S.Y.L.M.Y.X.Y. On the Chemical Constituents From the Flowers of *Dendrobium aurantiacum* Rchb. f. var. *denneanum*. *Industrial Microbiology* **2023**, *53*, 14-16.
101. YANG Dan , C.Z.-q., DING Zhong-tao , ZHOU Jun , HU Jiang-miao. Phenolic chemical constituents of *Dendrobium aphyllum*. *Chinese Traditional and Herbal Drugs* **2017**, *48*, 2839-2842.
102. ZHANG Xuel, G.H., WANG Nai-li, YAO Xin-sheng. Phenolic components from *Dendrobium nobile*. *Chinese Traditional and Herbal Drugs* **2006**, *37*, 652-655.
103. Yan Sha, M.R.-j., Yang Liu, Li Jin-yu, Yang Xiao-bei, Hu Jiang-miao. Chemical constituents and skin caring activities of *Dendrobium loddigesii*. *Natural Product Research And Development* **2019**, *31*, 615-620.

104. Zhang guangnong, Z.c., Luo ying, Wang zhengtao, Xu luoshan. Chemical Constituents of *Dendrobium thyrsiflorum* Rchb. f（II）. *CHINESE JOURNAL OF NATURAL MEDICINES* **2005**, *3*, 287-290.
105. Luo Dan, Z.C.-F., Lin Ping, Wang Zheng-Tao, Xu Luo-Shan. Study on chemical constituents of *Dendrobium nobile*. *Chinese Traditional and Herbal Drugs* **2006**, *37*, 36-38.
106. Zheng We-Iping, T.Y.-P., Lou Feng-Chang, Zhi Fei. Studies on the Constituents of *Dendrobium chryseum* Rolfe. *Journal of China Pharmaceutical University* **2000**, *31*, 5-7.
107. Bi Zhi-Ming, M.J.-F., Zhu Lin, Wang Zheng-Tao, Xu Luo-Shan. Study on Chemical Constituents of *Dendrobium aduncum*. *Chinese Pharmaceutical Journal* **2006**, *41*, 1618-1620.
108. Tianhong, N.J.C.J.H.C.L.H.C. Study on hypoglycemic activity and chemical constituents from *Dendrobium chrysanthum* Lindl. *Journal of Guangdong Pharmaceutical University* **2015**, *31*, 10-13.
109. Shu Ying, G.S.-X., Chen Xiao-Mei, Wang Chun-Lan, Yang Jun-Shan. Study on chemical constituents of *Dendrobium nobile*. *Chinese Pharmaceutical Journal* **2004**, *39*, 421-422.
110. Zhao Xin, Z.C.-F., Zhan Mian, Wang Zheng-Tao, Xu Luo-Shan. Study on Chemical Constituents from Stems of *Dendrobium crepidatum*. *Pharmaceutical and Clinical Research* **2011**, 136-138.
111. BI Zhi ming, W.Z.-t., XU Luo-shan, XU Guo-jun. Studies on the chemical constituents of *Dendrobium fimbriatum*. *Acta Pharmaceutica Sinica* **2003**, *38*, 526-529.
112. LI Yanru, C.X., XU Fengqing, WANG Renzhong, WU Deling. Anti-inflammatory and Cytotoxic Activity of *Dendrobium Huoshanense* C. Z. Tang et S. J. Cheng. *Research and Practice on Chinese Medicines* **2023**, *37*, 28-33.
113. Cai Bai-Xiang, S.L.-X., Hu Hai-Jun, Han Zhu-Zhen, Zhou Yue, Wang Zheng-Tao, Yang Li. Structures and biological evaluation of phenylpropanoid derivatives from *Dendrobium Sonia*. *Natural Product Research* **2020**, *35*, 5120-5124, doi:10.1080/14786419.2020.1782404.
114. HUANG Chujun, C.J., NI Jun, YANG Chao, ZHANG Tie. Study on chemical constituents from *Dendrobium chrysanthum* Wall. ex Lindl. *Journal of Guangdong Pharmaceutical University* **2016**, *32*, 279-281.
115. PAN Hongmei, C.B., LI Fu, WANG Mingkui. Chemical Constituents of *Dendrobium denneanum*. *Chinese Journal of Applied and Environmental Biology* **2012**, *18*, 378-380.
116. Xiao-Feng, L.J.-N.W.S.-S.D. Current Research Status of Chemical Constituents and Pharmacological Activity of *Dendrobium chrysotoxum* and *Dendrobium fimbriatum*. *Food and Nutrition in China* **2020**, *26*, 42-49.
117. ZHUANG Chenxi, W.H., CHEN Yaping, ZHANG Rui, ZHENG Jinrong, LI Meihong, LI Yupeng. Chemical Compounds Isolated from *Dendrobium crepidatum* and Their Bioactive Activities. *Journal of Kunming Medical University* **2023**, *44*, 1-5.
118. Ming Hu, Y.X.-Q., Zhou Qing-Yan, Li Shu-Quan, Wang Bang-Yan, Ruan Bao-Hui, Yang Ya-Bin, Zhang Zhuo-Xi, Zhou Hao , Ding Zhong-Tao. Benzopyran derivatives from endophytic Daldinia eschscholzii JC-15 in *Dendrobium chrysotoxum* and their bioactivities. *Natural Product Research* **2017**, *33*, 1431-1435, doi:10.1080/14786419.2017.1419236.
119. Wu Lei-lei, L.Y., Ding Yu-lian, Zhao Ju-run, Xu Hong, Chou Gui xin. Four new compounds from *Dendrobium devonianum*. *Natural Product Research* **2018**, *33*, 2160-2168, doi:10.1080/14786419.2018.1490900.

120. PAN Hongmei, C.B., LI Fu, WANG Mingkui. Chemical Constituents from the Stems of *Dendrobium denneanum* (II). *Chinese Journal of Applied and Environmental Biology* **2013**, *19*, 952-955.

121. Feng Yu, D.X.-D., Chen Hong-Yu, Duan Tie, Zhang Zai-Qiang, Yu Xiao-Ping, Zhang Wei-Wei. Research progress on chemical constituents and efficacy of *Dendrobium denneanum*. *Journal of Chengdu Medical College* **2015**, *10*, 612-615.

122. YANG Yuling, H.Y., LI Ling, CAI Qunhu, GUO Xibing, TIAN Yingqiu. Research Progress of *Dendrobium aurantiacum* Rchb. var. *denneanum* (Kerr) Z.H.Tsi. *CHINESE JOURNAL OF TROPICAL AGRICULTURE* **2020**, *40*, 50-58.

123. Zhang You-yuan, S.X.-q., Mei Wen-li, Zuo Wen-jian, Cai Cai-hong, Cheng Jin, Dai Hao-fu. Chemical Constituents from *Dendrobium hainanense* (Orchidaceae) in Hainan. *Journal of Tropical and Subtropical Botany* **2015**, *23*, 317-322.

124. Yang Hong, W.Z.-t., Xu Luo-shan, Hu Zhi-Bi. Chemical Constituents of *Dendrobium chrysotoxum*. *Journal of Chemical Pharmaceutical University* **2002**, *33*, 367-369.

125. Cai Jin-Yan, N.J., Chen Tian-Hong, Zhang Tie. A new phenanthrene from *Dendrobium chrysanthum*. *Chinese Traditional and Herbal Drugs* **2017**, *48*, 1506-1508.

126. Wang Xiao-Ya, M.C.-W., Zhou Qin-Mei. Research progress of sesquiterpenoids from *Dendrobium nobile*. *Natural Product Research And Development* **2019**, *31*, 1837-1845.

127. Bi zhiming, W.z., Xu luoshan. Chemical Constituents of *Dendrobium moniliforme*. *Acta Botanica Sinica* **2004**, *46*, 124-126.

128. Li An-Hua，Zhou Zhi-Hong，Shen Yan, Y.Y.-W. Chemical Constituents from Stem of *Dendrobium wardianum*. *Natural Product Research And Development* **2012**, *24*, 479-480.

129. Chen Ya-Ping, L.Y.-P., Zhang Zhong, Zhang Jun, Zhang Kai-Li, Huang Rong,Wen Xiao-Ling. Chemical Constituents from the Flowers of *Dendrobium thyrsiflorum*. *Journal of Kunming Medical University* **2016**, *37*, 5-7.

130. WENG Rui- xuan, L.Y.-p., CHEN Li- jun, HUANG Rong, TAO Yong- sheng , WANG Juan, LI Shuang, WEN Xiao- ling , ZHANG Gui- hua. Chemical Constituents from the Herbs of *Dendrobium Chrysanthum*. *Journal of Kunming Medical University* **2017**, *38*, 6-9.

131. Chen yaping, W.y., Li yupeng, Zhang jun, Weng ruixuan, Yang xiaolin. Research of Chemical Constituents from *Dendrobium Chrysotoxum*. *Asia-Pacific Traditional Medicine* **2015**, *11*, 14-15.

132. Dan Behr, K.L. Three steroid glycosides of the stigmastane type from *Dendrobium ochreatum*. *Phytochemistry* **1976**, *15*, 1403-1406.

133. Li Yan, W.C.-l., Wang Fang-fei, Dong Hai-ling, Guo Shun-xing, Yang Jun-shan, Xiao Pei-gen. Chemical constituents of *Dendrobium candidum*. *China Journal of Chinese Materia Medica* **2010**, *35*, 1715-1719.

134. Zhou XM, Z.C., Wu JT , Chen GY, Zhang B, Sun CG A new phenolic glycoside from the stem of *Dendrobium nobile*. *Natural product research* **2017**, *31*, 1042-1046.

135. Gong yanqing, Y.h., Liu yun, Liang aiqun, Wang zhengtao, Xu luoshan, Hu zhibi. Studies on chemical constituents in stem of *Dendrobium chrysotoxum*. *China Journal of Chinese Materia Medica* **2006**, *31*, 304-306.

136. Guogang, L.M.C.Y.D.B.S.S.Z.C.Z. Isolation and identification of chemical constituents from *Dendrobium officinale* Kimura et Migo. *Journal of Shenyang Pharmaceutical University* **2018**, *35*, 739-749.

137. chaofan, W. Studies on the chemical constituents of *Dendrobium nobile* Lindl. Yunnan Normal University, 2023.
